# Mandatory CSR and sustainability reporting: economic analysis and literature review

**DOI:** 10.1007/s11142-021-09609-5

**Published:** 2021-07-29

**Authors:** Hans B. Christensen, Luzi Hail, Christian Leuz

**Affiliations:** 1 University of Chicago, Chicago, IL USA; 2 University of Pennsylvania, Philadelphia, PA USA; 3 University of Chicago & NBER, Chicago, IL USA

**Keywords:** F30, G38, K22, L21, M14, M41, M48, Transparency, Regulation, SASB, GRI, Standard setting, Accounting standards, Mandatory disclosure, Environmental, social and governance issues (ESG)

## Abstract

This study collates potential economic effects of mandated disclosure and reporting standards for corporate social responsibility (CSR) and sustainability topics. We first outline key features of CSR reporting. Next, we draw on relevant academic literatures in accounting, finance, economics, and management to discuss and evaluate the potential economic consequences of a requirement for CSR and sustainability reporting for U.S. firms, including effects in capital markets, on stakeholders other than investors, and on firm behavior. We also discuss issues related to the implementation and enforcement of CSR and sustainability reporting standards as well as two approaches to sustainability reporting that differ in their overarching goals and materiality standards. Our analysis yields a number of insights that are relevant for the current debate on mandatory CSR and sustainability reporting. It also points scholars to avenues for future research.

## Introduction and outline of analysis

In 2019, the Business Roundtable issued a new statement of purpose for corporations and with a few words made a radical shift. For more than two decades, the group of top executives held the view that companies’ managers and directors had a primary duty to serve shareholders, but the updated statement now also lists customers, employees, suppliers, as well as supporting communities, promising a fundamental commitment to all stakeholders (Business Roundtable 2019). These executives are responding to mounting pressure that a company needs to do “good” while doing business, whether that means keeping carbon emissions low, waterways clear, or workers healthy. This shift on the corporate side is closely related to a growing desire by many to invest sustainably (e.g., Eurosif 2018; BlackRock 2020). For instance, in 2020, $17.1 trillion—or 33%—of assets managed in the United States (U.S.) were branded as sustainable investments (US SIF 2020).

Along with the growing interest in sustainable investments, the demand for information about corporate social responsibility (CSR) as well as firms’ environmental, social, and governance (ESG) activities and policies has steadily risen (Cohen et al. 2015; Amel-Zadeh and Serafeim 2018). Responding to this demand, 83% of companies registered with the U.S. Securities and Exchange Commission (SEC) disclose some sustainability information in their regulatory filings (SASB 2017c). However, much of this information is considered voluntary. It is therefore perhaps not surprising that investors complain about a lack of comparable and verifiable information (Bernow et al. 2019). In addition, the Sustainability Accounting Standards Board (SASB) points out that about 50% of SEC-registered companies provide generic or boilerplate sustainability information in their regulatory filings (Christensen et al. 2018; SASB 2017c).

In response to the demand for information and to the current state of corporate disclosure, numerous organizations offer (voluntary) reporting standards for ESG activities that aim to improve or harmonize reporting practices. For instance, the SASB develops “industry-specific disclosure standards across financially material environmental, social and governance topics” that firms can use in SEC filings. Similarly, the GRI (Global Reporting Initiative) strives to help companies “communicate their impact on critical sustainability issues” by developing global standards for sustainability reporting. The latest player to enter the fray is the IFRS Foundation, which is proposing a global approach to sustainability reporting to address the proliferation of standards and standard setters (IFRS 2020).^1^

At the same time, many jurisdictions are considering reporting mandates. In the U.S., the SEC’s Investor Advisory Committee has called for requiring SEC registrants to provide information related to ESG issues that is material to investors in their investment and voting decisions (IAC 2020; Coates 2021). The European Union (EU) is further along. Its Non-Financial Reporting Directive (NFRD 2014/95/EU) requires large companies and groups with more than 500 employees to provide “non-financial and diversity information” in the management report, starting in 2017. The NFRD adopts a double materiality perspective, stipulating that companies not only disclose how sustainability issues affect them, but also how their activities affect society and the environment. The EU is currently reviewing this directive and considering ways to strengthen it—for instance, by imposing particular standards or audit requirements (EC 2020).

What many of the aforementioned standard setting and regulatory initiatives have in common is that they see sustainability reporting as a critical ingredient for achieving broader climate and sustainability goals. Nevertheless, they differ widely in their motivations. Broadly speaking, extant regulatory approaches fall along a spectrum between two extremes characterized as follows. The narrow approach aims to “give investors what they want” and focuses on investors’ information needs. The key criterion here, as in financial reporting, is whether the information is material to investors when they make decisions and, hence, whether ESG issues could have financial consequences for the firm. The other approach addresses a broad audience, in principle all stakeholders or society, as it aims to “drive change” with sustainability reporting. The underlying idea is that reporting and the resulting transparency are change agents, incentivizing desirable behaviors and discouraging undesirables ones. The broad approach applies double materiality as the key criterion; that is, a firm not only reports how it is affected by ESG issues, but also the firm’s impacts on the environment and society, including the externalities it causes. By definition, the latter are not presently borne by the firm and therefore may not be material to investors.

The two approaches blend, once we recognize that investors can have preferences beyond shareholder value maximization and that “giving investors what they want” can entail information about a firm’s environmental or societal impacts. Moreover, ESG information that is provided to investors under the narrow approach can also be used by other stakeholders to assess corporate impacts on the environment and society. Thus, the two approaches may be less distinct in practice and in their consequences than they are at the conceptual level.

We suspect that the idea to “drive change” with respect to CSR and sustainability via reporting is popular in part because a reporting mandate is often viewed as less intrusive than traditional regulation, as the former merely prescribes disclosure whereas the latter compels particular actions. Moreover, pushing for more transparency is often politically expedient. Yet, mandatory disclosure regimes can have unintended consequences and are far from being innocuous (see, e.g., Dranove and Jin 2010 and Leuz and Wysocki 2016 for further discussion and examples). It follows that CSR and sustainability reporting regimes require careful economic analysis.

In this study, we take a first step towards an economic analysis of CSR and sustainability reporting (which should not be confused with an analysis of the CSR activities and policies themselves). To frame the analysis, we consider the adoption of a reporting mandate for U.S. publicly listed corporations.^2^ Our analysis is informed by an extensive review of the relevant academic literatures in accounting, finance, management, and economics. We apply insights from these literatures to derive and discuss possible economic consequences of a CSR reporting mandate, including potential capital-market effects, effects on other stakeholders, real effects, as well as implementation issues.

In our discussion, we consider potential consequences from reporting financially material ESG information as well as from disclosing information about corporate impacts on the environment and society. Doing so allows us to discuss a wide array of issues and potential consequences. But we also come back to the tradeoffs in choosing between a narrow approach to CSR reporting that focuses on investors and financial (or single) materiality versus a broader approach of informing stakeholders about corporate impacts (with double materiality).

We use the terms “CSR” and, interchangeably, “sustainability” activities and policies to denote corporate actions that assess, manage, and govern a firm’s responsibilities for and impacts on society and the environment.^3^ CSR often has the goal of improving social welfare or making business activities more sustainable. CSR goes beyond compliance with legal, regulatory, and contractual requirements (McWilliams and Siegel 2001), and in this sense CSR activities and policies are often voluntary, although they can be strategic or induced by markets (Kitzmueller and Shimshack 2012). CSR could be in line with or go against the interests of shareholders. It encompasses a broad spectrum of environmental, social, and governance topics, activities, and policies. In line with this definition, we refer to CSR and sustainability *reporting* as the measurement, disclosure, and communication of information about CSR or ESG topics, activities, risks, and policies and use the shorthand “CSR standards” to refer to a mandate for reporting on CSR or sustainability issues (e.g., a requirement to follow a specific set of reporting standards).

We organize our economic analysis as follows. Section 2 outlines the scope of the analysis and provides the conceptual underpinnings. We provide definitions for the key terms (Section 2.1) and delineate the primary scenario for our analysis, that is, the mandatory adoption of CSR reporting standards for U.S. public firms (Section 2.2). We contrast this scenario to the status quo of voluntary CSR reporting, which allows us to highlight the potential economic effects of a CSR and sustainability reporting mandate. Next, we point out key conceptual features that distinguish CSR and sustainability reporting from a traditional financial reporting system (Section 2.3). We conclude with a brief discussion of general insights from extant academic literature in accounting, finance, management, and economics (Section 2.4). For instance, we stress the importance of firm-level incentives and institutional complementarities in shaping firms’ reporting practices and regulatory outcomes. We later apply these general insights to CSR disclosures and reporting.

Section 3 outlines key determinants of firms’ voluntary CSR reporting decisions. Many U.S. firms currently provide CSR information either on a voluntary basis or because they deem the information material to investors under existing securities law. As such, voluntary disclosure practices provide insights into when firms are more likely to find CSR reporting beneficial. We first outline generic firm and manager characteristics associated with (voluntary) CSR reporting that prior literature has identified (Section 3.1). Next, we review the role of business activities and environmental events in shaping firms’ CSR disclosures (Section 3.2). Pressures from outside stakeholders and society can also play a role in shaping CSR reporting practices (Section 3.3). We conclude with a discussion of the implications of these determinants and the current state of (largely voluntary) CSR reporting in the U.S. for our analysis (Section 3.4).

Section 4 discusses the potential effects of mandating CSR and sustainability reporting standards on the various recipients of these disclosures. We begin by briefly reviewing the link between CSR activities and shareholder value or firm performance (Section 4.1). This link is central to the CSR literature and one of the key motives for why investors demand information about CSR and sustainability issues. We then outline potential capital market effects, first focusing on equity investors (Section 4.2) and then on debtholders or lenders (Section 4.3). These two groups are typically viewed as the main users of financial statements. We discuss effects of CSR reporting on firm value, stock returns, liquidity, risk, and cost of capital, as well as on investors’ portfolio holdings. Next, we turn to financial intermediaries, including analysts and the business press, both of which play a role in processing and disseminating CSR information (Section 4.4). Finally, we analyze potential effects of CSR and sustainability reporting on other stakeholders without a direct financial claim on the firm, such as customers, management, employees, and the society at large (Section 4.5). We conclude the section with a discussion of what we learn more generally about the effects of a CSR reporting mandate on various stakeholders (Section 4.6).

In Section 5, we consider potential firm responses to and real effects from mandatory adoption of CSR reporting standards. Such consequences likely arise irrespective of whether the mandate aims to inform investors or explicitly intends to drive change with respect to sustainability. Reporting mandates often induce firms to alter their behavior precisely because investors or other stakeholders respond to firm disclosures (or are expected to do so). We briefly review the link between disclosure (regulation) and firms’ real investment, financing, and operating activities (Section 5.1). We then discuss what we know about the potential firm-level consequences of forcing firms to provide CSR information, and we review extant literature on the real effects of CSR and sustainability reporting (Section 5.2). Next, we consider the possibility that firms abandon certain business activities to avoid controversial CSR and sustainability issues or exit certain markets altogether once they face CSR reporting regulation (Section 5.3). Some of these adjustments are intended, but others may be unintended. We conclude by discussing potential implications of a CSR reporting mandate for aggregate CSR activity in the economy (Section 5.4).

In Section 6, we turn to key implementation issues that arise when mandating CSR and sustainability reporting. While many of these issues arise in accounting standard setting in general, we incorporate the distinctive features of CSR reporting into the analysis. Specifically, we discuss the process of establishing and maintaining CSR reporting standards (Section 6.1); the role of (single or double) materiality for CSR reporting, including a review of the empirical evidence on the effects of material CSR disclosures (Section 6.2); the use of boilerplate language by firms to achieve compliance with CSR standards or to mask poor CSR performance (Section 6.3); and challenges for an effective enforcement of CSR reporting standards, including the role of assurance providers for the certification of CSR reports (Section 6.4).

Section 7 concludes with a summary of the main insights from our analysis. We point to several open questions as well as areas that are currently under-researched. As such, our discussion highlights numerous opportunities for future research in the area of CSR, ESG, and sustainability reporting.

It is important to note that our analysis does not evaluate the potential costs and benefits of CSR activities or policies themselves. The focus is on the economic effects of a mandate for CSR and sustainability reporting. We do not consider the pros and cons of specific CSR standards, nor do we analyze individual standards for particular industries or activities. Our goal is to identify key economic tradeoffs and important economic effects that can be reasonably expected from a mandate, considering the status quo of corporate reporting and extant evidence. Predicting economic consequences of a CSR and sustainability reporting mandate is difficult and fraught with uncertainty, just as it is for any other policy change. Thus, we consider our analysis—in parts—as speculative (see Section 2.2 for additional caveats and limitations).

## Scope of analysis and conceptual underpinnings

We first define key terminology (Section 2.1) and delineate the scope of our study (Section 2.2). We then highlight the conceptual features that distinguish CSR reporting from financial reporting (Section 2.3). We conclude with a brief discussion of relevant insights from the academic literature in accounting, finance, management, and economics pertaining to mandatory disclosure, reporting standards, and international accounting (Section 2.4), which we later apply to CSR reporting.

### Key definitions

Our review of definitions for the two terms “CSR” and “sustainability” indicates that their meanings are close and that they are often used similarly. CSR tends to be defined slightly more broadly and normatively; sustainability, in turn, emphasizes the long-term horizon. While the use of the term sustainability by companies is growing (e.g., KPMG 2013, p. 6), CSR is still the predominant term in the academic literature (Huang and Watson 2015). We use CSR and sustainability interchangeably, but mainly use the acronym CSR for brevity.

We define CSR as corporate activities and policies that assess, manage, and govern a firm’s responsibilities for and its impacts on society and the environment. CSR often has the goal of improving social welfare or making corporate activities more sustainable. CSR could be fully in line with the interests of shareholders and even increase the value of the firm (e.g., by building trust and social capital; Lins et al. 2017). However, maximizing firm value is not necessarily the same as maximizing shareholder welfare (Hart and Zingales 2017). Shareholders could have non-monetary preferences and, hence, care about a company’s (negative) impact on the environment or society even when this impact does not have (immediate) financial consequences (as is the case with externalities such as CO_2_ emissions). CSR often implies that a firm pursues a broader objective than maximizing its market value, trying to meet the needs and expectations of a wider set of stakeholders or society. In doing so, firms may sacrifice profits (e.g., Roberts 1992; Bénabou and Tirole 2010), although the absence of profit or shareholder value maximization is not a necessary feature of the CSR definition (e.g., Kitzmueller and Shimshack 2012).^4^ CSR goes beyond compliance with legal, regulatory, and contractual obligations, highlighting its voluntary nature (e.g., McWilliams and Siegel 2001; Liang and Renneboog 2017), and also extends beyond having good corporate governance. It encompasses a wide range of environmental, social, and governance (ESG) activities (or policies) without sharp boundaries.^5^

Based on the above definition, we refer to CSR *reporting* as the measurement, disclosure, and communication of information about CSR and sustainability topics, including a firm’s CSR/ESG activities, risks and policies. CSR *reporting standards*, in turn, govern how to report and disclose such information. Firms can include CSR information in their annual report or provide a separate CSR report, sometimes referred to as a sustainability, corporate accountability, or nonfinancial report.^6^ CSR reports (or the relevant sections in the annual report) contain a broad range of qualitative and quantitative, but not necessarily monetary, information. Firms may ask an auditor, consultant, or another external assurance provider to certify their CSR reports and disclosures (e.g., Sìmnett et al. 2009; Casey and Grenier 2015).

An important dimension of CSR reporting and standards is their scope, both in terms of the breadth of the reported information and the intended audience. Conceivable approaches fall along a spectrum between two extremes. Under the narrow approach, CSR reporting is confined to information deemed relevant or material for *investors* in their investment decisions. CSR information can be useful to investors in estimating future cash flows or assessing firms’ risks, because CSR and sustainability topics are often closely related to firms’ normal business activities (e.g., Dhaliwal et al. 2011; Dhaliwal et al. 2012; Grewal et al. 2020). For this reason, firms may already have to disclose certain CSR information under existing securities laws (see e.g., Wallace 1993; Grayson and Boye-Williams 2011). For example, it is unlawful for registrants with the SEC to omit material facts (Section 17(a)(2) of the Securities Act of 1933; Section 18(a) of the Securities and Exchange Act of 1934), regardless of whether or not this information pertains to CSR topics. Moreover, Item 303 of Regulation S-K requires firms, among other things, to “describe any known trends or uncertainties that have had or […] will have a material favorable or unfavorable impact on net sales or revenues or income from continuing operations,” again making no distinction about whether or not these trends or uncertainties pertain to CSR topics. Thus, on one end of the spectrum, CSR reporting is limited to information that is *financially* material to investors for their decision making, and it might not go much beyond existing U.S. reporting requirements. This narrow approach still covers many CSR topics, but it excludes reporting about externalities for which the firm receives some benefits but does not bear the full costs.

On the other end of the spectrum is the broad approach to CSR reporting. It expands the scope and target audience of CSR reporting and provides information that is relevant (or material) to a diverse set of stakeholders, such as consumers, employees, or local communities. Under this stakeholder-oriented approach, firms also report information about their impacts on the environment and society, irrespective of whether these impacts have financially material consequences for the firm. Thus, this approach includes reporting about externalities (e.g., CO_2_ emissions that are not covered by an emission pricing regime). As this approach may still subsume the information that is material to investors, it is referred to as having a double materiality criterion.

In our analysis, we consider the economic consequences from both more narrowly reporting financially material CSR information aimed at investors (single materiality) and more broadly disclosing information about corporate impacts on the environment and society to multiple stakeholders (double materiality). This inclusive perspective is in line with much of extant CSR literature, which does not necessarily make the distinction between the two types of information. Moreover, considering both sets of CSR information allows us to discuss a broader array of issues and potential consequences. We also recognize that investors often have non-monetary preferences and are part of other stakeholder groups, such as consumers or employees; hence, they may care about CSR information even when it is not financially material to the firm. In Section 6.2.2, we come back to this distinction and discuss to what extent key insights change when the CSR reporting regime focuses on the information that is material to investors.

### Framing the scope of the analysis: mandatory adoption of CSR reporting standards

To frame the analysis, we consider the adoption of a mandatory CSR reporting regime with CSR reporting standards for public U.S. companies. This regime would not be limited to certain industries or types of firms but would apply to all corporations that file with the SEC, although the individual standards could be industry specific. We focus on U.S. companies to limit the analysis to one institutional environment, but we believe that much of our discussion and analysis applies to firms in other developed economies (e.g., EU countries). We consider a mandate rather than a further increase in voluntary CSR reporting for several reasons. First, the current policy debate in the U.S. revolves largely around the question of a mandate that explicitly imposes CSR reporting requirements on companies. In the EU, such a mandate already exists (NFRD 2014/95/EU). Moreover, to analyze voluntary reporting changes (e.g., firms individually deciding to follow a specific set of CSR standards), we would have to consider firms’ adoption decisions, the effects on non-adopters, as well as coordination problems across firms in terms of adoption timing and what standards to follow. Doing so would complicate the discussion considerably because voluntary adoption decisions depend on firms’ *private* costs and benefits, which likely differ across firms.

By *mandatory* CSR reporting, we mean the adoption of formal CSR reporting standards that prescribe (i) what firms have to report about their CSR activities, risks, and policies, (ii) which CSR topics are relevant for certain industries and firms, (iii) which metrics are important and how they are computed, and (iv) where and how the information is to be presented. That is, the standards provide structure and guidance for firms’ CSR reporting, including definitions of materiality thresholds, information content, and reporting formats.

CSR standards could be stand-alone or embedded in existing SEC requirements (see SEC Concept Release 2016). They could be set by the SEC, by another governmental entity, or by an independent standard setter following the model for financial reporting. We do not discuss these finer distinctions and neither endorse a particular standard setter nor consider a specific set of standards (e.g., SASB, GRI). The baseline for the analysis is the status quo of current CSR reporting by U.S. firms. That is, we assume that without a mandate, firms continue to provide CSR information, either voluntarily or by following the existing SEC disclosure requirements (e.g., Regulation S-K or Section 1502 of the Dodd-Frank Act).^7^ We consider CSR standards as ostensibly stipulating more (or higher-quality) CSR reporting and/or more comparable CSR reporting, which in turn can also be more informative, relative to the status quo. An important question then is whether CSR *standards* by themselves are likely to achieve better and more comparable CSR reporting in *practice*. We discuss this question at a conceptual level in Section 2.4, as it naturally comes before assessing the economic effects of CSR reporting.

The scope of our analysis is limited in several ways. First, we focus primarily on the effects of *reporting* about firms’ CSR activities, and not on the CSR activities themselves. Thus, we do not consider issues such as why firms engage in CSR activities in the first place (Ramirez 2013; Borghesi et al. 2014; Windolph et al. 2014), whether CSR activities are positive net present value (NPV) projects (e.g., Flammer 2015; Khan et al. 2016; Manchiraju and Rajgopal 2017) or provide non-monetary payoffs to some investors (e.g., Fama and French 2007; Friedman and Heinle 2016; Martin and Moser 2016), whether CSR activities require a long-term investment horizon and serve to mitigate managerial myopia (e.g., Stein 1989; Bénabou and Tirole 2010), what the optimal level of CSR activities is (e.g., McWilliams and Siegel 2001), or why CSR activities vary across firms, industries, and countries (e.g., Wickert et al. 2016; Liang and Renneboog 2017). But we realize that the effects of CSR reporting are invariably linked to the underlying CSR issues and activities.

Second, we do not (and cannot) conduct a comprehensive cost-benefit analysis. We do not attempt to quantify or qualitatively evaluate the *net* benefits (or costs) of a CSR reporting mandate for individual firms or society as a whole. However, by highlighting potential economic effects and tradeoffs, we hope that our study provides structure for researchers, standard setters, and policymakers when they evaluate the consequences of a CSR reporting mandate.

Third, although governance issues—the letter “G” in ESG—are an integral part of CSR activities and initiatives and are included in our CSR definition (Section 2.1), we do not explicitly discuss governance as a means to achieve CSR goals or increase CSR disclosures in our study. The economic effects of governance mechanisms are well documented in the literature, and firms provide extensive (mandatory) disclosures on governance issues. We therefore refer the reader to the specific literature (e.g., see Armstrong et al. 2010, for a review).

Finally, while we draw extensively on academic (CSR) research in accounting, economics, finance, and management, the goal in this study is not to provide a comprehensive survey of the CSR literature.^8^ Nevertheless, we incorporate relevant papers that were published in the top accounting and finance journals up to the year 2020 and, more selectively, papers published in top economics and management journals. In addition, our earlier research report (see Christensen et al. 2018), which formed the basis for this study, comes with an Online Appendix that provides an extensive overview and brief summary of extant CSR research (as of May 2017; available at: http://ssrn.com/abstract=3313793). This Appendix contains a summary of over 380 CSR studies, which we identified in a systematic literature search. For each study, the summary briefly delineates the research question and the research design (including the variables of interest) and indicates the results in tabular form. We cross-reference this Online Appendix throughout the paper.

### Key features of CSR reporting relative to financial reporting

CSR encompasses a wide range of ESG topics, which could be indirectly or only tangentially related to firms’ core operations. As a result, CSR reporting differs from traditional financial reporting, which deals with the financial implications of firms’ main and regular business activities. The following key features are integral to CSR reporting:

1. *Diversity of users and uses:* The potential audience of CSR reporting is broader than for financial reporting. Even when the standards are developed with the needs of investors in mind, once CSR information is disclosed, anyone can use it. The same is of course true for financial reporting, but for CSR information the users may include groups that have relatively little experience in reading corporate disclosures (e.g., consumers). Moreover, these groups could use CSR information for a variety of purposes beyond traditional financial analysis—for instance, to evaluate a firm’s broader contribution to society or whether a firm adheres to policies that are consistent with specific norms and ethical values.
2. *Diversity of topics*: As CSR and sustainability are not sharply defined, they encompass a broad range of ESG topics, activities, and policies. The topics differ substantially across firms, industries, and countries. As a result, CSR reporting is multidimensional in nature, which leads to a broad variety of disclosures and reporting formats and makes comparisons and standardization difficult (Kitzmueller and Shimshack 2012; Liang and Renneboog 2017; Amel-Zadeh and Serafeim 2018).
3. *Diversity of objective functions:* CSR reporting has many objectives and responds to a wide range of interests and preferences from within and outside the firm. These interests and preferences can change quickly over time, as, for instance, when the firm becomes the target of a social activist campaign (e.g., Baron 2001), or as a result of exogenous events like an accident or natural catastrophe (e.g., Blacconiere and Patten 1994; Bonetti et al. 2018).
4. *Diversity in measurement:* Many CSR activities manifest in observable and measurable behaviors or (technical) outputs (e.g., CO_2_ emissions, number of trees saved), but they are not necessarily measurable in monetary terms (Kitzmueller and Shimshack 2012). When there is little uniformity, it is difficult to apply typical accounting conventions, such as double-entry bookkeeping or basic accounting principles like materiality, matching, and relevance to CSR reporting (e.g., Cohen and Simnett 2015; Moroney and Trotman 2016).
5. *Voluntary nature of CSR activities:* In most instances, CSR activities and policies are voluntary in nature and go beyond legal, regulatory, and contractual requirements.^9^ For instance, a firm may reduce pollution beyond what is required by law or provide a public good to the local community. As a result, what firms have to report under a mandate is a function of their underlying CSR choices (or lack thereof).^10^
6. *Long-term horizon:* CSR is often viewed as a “strategic” activity that foregoes short-term profits in return for long-term benefits to the firm (e.g., Bénabou and Tirole 2010). Thus, CSR reporting frequently has to deal with long-term prospects that are difficult to quantify and intangible in nature (e.g., consumer goodwill or employee relations).
7. *Central role of externalities:* CSR often pertains to externalities—that is, companies’ impacts on the environment and society—with the classic example being CO_2_ emissions. Addressing such externalities is one of the primary motivations for a CSR reporting regime using double materiality. Moreover, CSR reporting can extend to topics and activities beyond the traditional boundaries of the firm (e.g., when a firm imposes child labor restrictions on its supply chain).

These key features of CSR reporting predict considerable heterogeneity in firms’ reporting practices. Thus, the standardization of CSR reporting through mandatory CSR standards could yield substantial benefits. But the very same features pose significant challenges for measurement, comparability, and standardization on its own but also relative to financial reporting. The above features of CSR reporting therefore imply that it is harder to predict the economic consequences of a CSR reporting mandate, compared to the effects of financial reporting standards.

### Conceptual underpinnings and general insights from extant literature

We discuss several general insights about disclosure and financial reporting that are relevant when contemplating a CSR reporting mandate. First, we highlight the main economic effects of corporate disclosure and reporting, mostly in capital markets (Section 2.4.1). Next, we discuss the role of regulation in improving and harmonizing financial reporting *practices* (Section 2.4.2). We highlight the importance of firm-level incentives and institutional complementarities in shaping reporting practices and regulatory outcomes (Section 2.4.3). Finally, we point to enforcement as a central element in the effective implementation of reporting regulation (Section 2.4.4).

#### Economic effects of corporate disclosure and reporting

A primary benefit of corporate disclosure is to mitigate information asymmetries between the firm and its investors as well as among investors.^11^ Corporate disclosure can play several roles in this setting. First, disclosure can mitigate the adverse selection problem and level the playing field among investors (Verrecchia 2001), which in turn should increase the liquidity of secondary securities markets and lower the return that investors require for investing in firm stock (e.g., Constantinides 1986; Amihud and Mendelson 1986). Second, disclosure can make it easier for investors to estimate future cash flows and covariances between them, lowering the cost of capital (e.g., Easley and O’Hara 2004; Lambert et al. 2007; Lambert et al. 2011). Third, disclosure can raise investor awareness or their willingness to hold securities, which improves risk sharing in the economy (Merton 1987; Diamond and Verrecchia 1991). Fourth, disclosure facilitates the monitoring of managers by corporate outsiders such as analysts or institutional investors, thereby improving managerial decision making and leading to more efficient corporate investments (e.g., Bushman and Smith 2001; Lambert et al. 2007). Finally, disclosure by one firm can provide useful information about other firms in the form of information transfers and spillovers (e.g., Foster 1980; Dye 1990; Admati and Pfleiderer 2000), and such externalities could be an important reason for mandating disclosure and reporting (e.g., Bushee and Leuz 2005).

The general takeaway from this large literature is that more and better disclosure can lead to tangible capital-market benefits in the form of improved liquidity, lower cost of capital, higher asset prices (or firm value), and potentially better corporate decisions.^12^ There is also substantial empirical evidence consistent with these links and effects, though the strength of the evidence differs by economic construct or outcome (e.g., Healy and Palepu 2001; Leuz and Wysocki 2016). To the extent that mandatory CSR reporting and CSR standards improve the information available to investors, the same theories and many of the prior findings should apply when considering the economic effects of the mandate or the standards.

Similar parallels apply on the cost side. Disclosure has direct and indirect costs, which could offset the aforementioned benefits. The direct costs include the preparation, certification, and dissemination of accounting reports. The indirect costs can occur in the form of proprietary costs, because multiple audiences (e.g., competitors, suppliers, labor unions, etc.) can use the information provided to investors (e.g., Verrecchia 1983; Feltham and Xie 1992; Berger and Hann 2007). Proprietary cost considerations are less relevant for high level or aggregated disclosures, but they can arise for fairly specific or detailed disclosures and especially for smaller firms (e.g., Bens et al. 2011; Leuz et al. 2008). More detailed reporting can also hurt firms’ innovation incentives (e.g., Breuer et al. 2020), which could be quite relevant in a CSR context. In addition, forward-looking disclosures, especially when too optimistic, could expose firms to higher litigation risk (e.g., Johnson et al. 2001; Rogers et al. 2011), while the disclosure of bad news could reduce the likelihood and costs of litigation (e.g., Skinner 1994, 1997; Field et al. 2005; Donelson et al. 2012). Finally, disclosure, especially when mandated, can have negative real effects, both from a firm’s and a societal perspective (e.g., Dranove et al. 2003). These effects stem from attempts to manage required disclosures through real actions and arise especially when the disclosure only poorly measures the quality of the underlying actions (Dranove and Jin 2010).

All these disclosure costs are not specific to financial reporting but also apply to the disclosure and reporting of CSR information. For instance, the multiple-audience issue from the proprietary cost literature is relevant for CSR information (see Section 5).

#### Regulation to improve and harmonize financial reporting

Proponents of mandatory disclosure regulation and accounting standards typically point to transparency and comparability benefits arising from standardized financial reporting.^13^ However, the existence of such benefits is not enough to justify a mandate; in the presence of net private benefits, firms have incentives to reveal information voluntarily.^14^ An economic rationale for regulation therefore requires the existence of positive externalities (or spillovers), market-wide cost savings from regulation, or dead-weight economic losses that mandated disclosure could mitigate.

Disclosure externalities arise when the public value of the disclosed information differs from the private value. It is quite plausible that disclosures by one firm can provide information about other firms (e.g., Foster 1980), resulting in positive externalities. However, firms may not consider these positive externalities from their reporting choices in the aggregate, which provides a rationale for creating reporting standards and mandating their use. In addition, regulation of reporting can provide market-wide cost savings when it reduces duplication in the production and acquisition of information. Generic disclosures that are relevant to all firms and many users are especially likely to generate such savings. Similarly, standardizing firms’ reporting practices can make comparisons across firms easier and less costly. Another argument is that privately producing a credible commitment to disclose can be expensive if not impossible. In this case, a mandate serves as commitment device (e.g., Mahoney 1995; Rock 2002) that forces firms to provide information regardless of the underlying news content (i.e., good or bad news). Finally, disclosure regulation can mitigate dead-weight losses to the economy. For instance, agency problems and private control benefits to corporate insiders often induce suboptimal investment behavior (Shleifer and Vishny 1997; Shleifer and Wolfenzon 2002). In this situation, disclosure regulation can facilitate capital raising by new entrants (e.g., offering commitment) so that they can exploit the opportunities left by incumbents, and reduce social losses.^15^ We can make a similar argument when firm behavior creates negative externalities and social costs (e.g., pollution). In this situation, disclosure regulation can serve to create or support a price mechanism (Hart 2009), which makes firms internalize the social costs and, hence, reduce activities with negative externalities.

The general takeaway from the literature is that arguments in favor of mandatory CSR standards need to articulate why and how a mandate reduces negative (or leads to positive) externalities, creates economy-wide cost savings, reduces existing dead-weight losses or social costs, or creates commitment that does not exist in a voluntary disclosure regime. That being said, the existence of cost savings and standardization benefits to the users of CSR information, a stronger commitment to disclosure, and the potential to mitigate negative externalities from firms’ business activities are reasonable arguments in support of a CSR mandate. However, it is important to recognize that mandatory disclosure regimes are costly to design, implement, and enforce, and it is not a priori obvious that they would necessarily achieve better outcomes or be cheaper than a market solution. For instance, regulators and standard setters can be captured by the regulated (e.g., Stigler 1971; Mahoney 2001). Moreover, firms are likely better informed about their cost-benefit tradeoffs with respect to corporate disclosure, suggesting that regulators face substantial information problems (e.g., Hail et al. 2018; Christensen et al. 2020).

There is considerable empirical work on the effects of reporting regulation, but this work also has important limitations (e.g., discussed in Leuz and Wysocki 2016). First, evidence on the causal effects of regulation is still scarce. Second, we typically lack evidence that allows us to perform quantitative cost-benefit analyses for a specific regulatory proposal or act. Third, new regulation does not occur in a vacuum, but interacts with other features of the institutional environment. Such relations often affect the effectiveness of regulation and can render the outcomes context specific. These caveats and challenges likely also apply to a mandate for CSR reporting.

#### Standards, incentives, and complementarities to shape reporting practice

Accounting and disclosure standards provide a framework, rules, and guidance for firms’ reporting practices.^16^ Yet, for good reasons, reporting standards contain discretion, and the application of the standards requires considerable judgement.^17^ Reporting discretion implies that other factors—not just the standards—determine reporting practices and outcomes, including managerial incentives and other institutional arrangements.

Managerial reporting incentives reflect many factors, such as a firm’s capital needs, managers’ compensation schemes, and competition in product markets. These incentives shape reporting behavior and influence the properties of reported accounting numbers and disclosures (e.g., Ball et al. 2000; Leuz et al. 2003; Burgstahler et al. 2006), including the extent to which firms manage earnings (e.g., Watts and Zimmerman 1990; Healy and Wahlen 1999; Dechow and Skinner 2000; Dechow et al. 2010). Thus, firm-level incentives are a source of substantial and predictable variation in reporting outcomes. Reporting standards and their proper enforcement can only partially mitigate this variation. Convergence in reporting practices requires that the underlying incentives be similar, which rarely is the case (e.g., Bradshaw and Miller 2008; Leuz 2010). Prior evidence supports these arguments and shows that differences in reporting outcomes persist even when firms use the same standards (e.g., Ball et al. 2003; Leuz 2003; Lang et al. 2006; Daske et al. 2013). This evidence is highly relevant in the context of CSR reporting standards.

Other institutional arrangements also impose constraints on what standards can achieve. There exist intricate complementarities among the many institutions in a market or country. That is, elements are chosen or designed so that they have institutional fit. Changes to one element (e.g., reporting standards) cannot be considered in isolation from the other elements (e.g., the litigation system), because such changes may make the entire system (or economy) worse off, even when they improve one element along a particular dimension. Thus, institutional fit should be part of the consideration when contemplating mandatory CSR reporting.

#### Importance of enforcement for effective reporting regulation

An enforcement mechanism is typically an integral part of any new regulation.^18^ It follows that the effectiveness of a CSR reporting mandate depends, among other things, on its enforcement. This insight is not new and is supported by evidence from settings such as the mandatory adoption of IFRS (e.g., Byard et al. 2011; Landsman et al. 2012; Christensen et al. 2013), the enactment of insider-trading laws (Bhattacharya and Daouk 2002), and the introduction of new securities regulation in the EU (Christensen et al. 2016).

A socially desirable level of enforcement can be achieved using various strategies involving market discipline, private litigation, public enforcement, or state ownership (e.g., Shleifer 2005). Based on the premise that all these strategies are imperfect, Djankov et al. (2003) formulate an enforcement theory of regulation that stipulates a mix among the various imperfect alternatives that minimizes social losses. Each strategy offers different pros and cons. From the viewpoint of this theory, regulation is particularly suited for situations in which the “inequality of weapons” between parties is large. Shleifer (2005) argues that this situation arises in the case of securities or disclosure regulation. Such an inequality likely exists for CSR reporting as well. Regulation is also more likely to be beneficial when the odds of public abuse of power are low, as they are in jurisdictions with strong checks and balances on government and regulatory agencies, such as the U.S. We come back to the enforcement issue for mandatory CSR reporting in Section 6.4.

## Key determinants of voluntary CSR reporting

We review key determinants of firms’ (voluntary) CSR reporting decisions, which shape the status quo. Observed disclosure practices provide insights into when firms are more likely to find CSR reporting beneficial, which in turn can be useful in understanding which firms would likely be more or less affected by a mandate. However, given that voluntary disclosure choices reflect firms’ private cost-benefit tradeoffs, this evidence only indirectly speaks to the benefits of a CSR reporting mandate (see Section 2.4.2). An important caveat of the evidence discussed in this section is that CSR reporting often has tight links to firms’ voluntary CSR activities and policies, and that studies typically cannot separate the two. Based on extant literature, we identify several firm and manager attributes (Section 3.1), firms’ business activities and external events (Section 3.2), as well as outside pressure by stakeholders and society (Section 3.3) as key determinants of voluntary CSR reporting. We then discuss the implications of these determinants and the current state of CSR reporting for a potential CSR reporting mandate (Section 3.4).

### Generic firm and manager characteristics

Academic studies have identified many firm attributes that are associated with the decision to disclose CSR information (see Table A1 in the Online Appendix). One of the most common findings is a significantly positive association between firm size and the quantity or quality of CSR disclosures (e.g., Hahn and Kühnen 2013; Li et al. 2021). This positive relation could be explained by greater public scrutiny of large firms, which arguably incentivizes them to engage in CSR activities *and* reporting (e.g., Cormier and Magnan 2003; Thorne et al. 2014). Another explanation may be that CSR communication is relatively less costly for larger firms, while the actual implementation of CSR activities is not (Wickert et al. 2016).

Ownership structure is another factor frequently associated with CSR disclosures. For instance, Höllerer (2013) finds a positive association between dispersed private-sector ownership and the decision to disclose stand-alone CSR reports. Similarly, Cormier and Magnan (1999) and Cormier et al. (2005) find that concentrated ownership is associated with less environmental disclosure. Teoh and Thong (1984) find that foreign ownership, particularly from the U.S. and U.K., is positively related to CSR reporting among a sample of Malaysian firms. These findings suggest that CSR reporting is more prevalent when information asymmetry is high or when firms need to communicate with a larger set of shareholders.

Another stream of literature examines the association of CSR disclosures with corporate governance structures and managerial characteristics. For example, Dalla Via and Perego (2018) find that various measures for the strength of firms’ corporate governance systems (e.g., long-term managerial incentive schemes, number of board meetings, etc.) are positively associated with CSR disclosures. Mallin et al. (2013) find that firms with a greater stakeholder orientation in their corporate governance policies also disclose more (and better) information on social and environmental issues. Regarding managerial characteristics, studies find associations between CSR reporting and managers’ educational levels and training (Lewis et al. 2014), personal views (e.g., Adams and McNicholas 2007; Parker 2014), ethnicity (Haniffa and Cooke 2005), whether the CEO has a daughter (Cronqvist and Yu 2017), (over-)confidence (McCarthy et al. 2017), and prior expertise with CSR issues (Peters and Romi 2015). Consistent with managerial characteristics playing an important role in firms’ CSR activities and reporting, Davidson et al. (2018) find that CEO fixed effects explain 59% of the variation in CSR scores, whereas firm fixed effects only explain 23%.

The finding that firm size, ownership, corporate governance, and management characteristics are associated with voluntary disclosures is not unique to CSR reporting. The same or similar attributes are also related with firms’ financial disclosures (Healy and Palepu 2001; Leuz and Wysocki 2008). These common associations suggest that there is significant overlap in the economic drivers of CSR reporting and of more traditional (non-CSR) disclosures.

### Firms’ business activities and external events

Research shows associations between firms’ economic activities and their CSR reporting. Firms in “polluting” industries tend to have higher levels of environmental disclosures (Gamerschlag et al. 2011). Similarly, studies suggest a link between how controversial an industry is in the public eye and the extent of its CSR reporting. For instance, Byrd et al. (2016) find that alcohol, tobacco, and firearm firms disclose more on social and community actions than firms in noncontroversial industries. The authors argue that these disclosures help firms legitimize their operations and convey to the public that they take actions to offset the perceived social problems with their business model. Grougiou et al. (2016) find that firms in so-called “sin” industries are more likely to provide CSR reports. They also find evidence suggesting that CSR reports lessen litigation risk stemming from firms’ controversial activities. Overall, the evidence is broadly consistent with the idea that CSR reporting is used to shape public opinion of firms’ impact on society.

Several studies examine the relation between performance of CSR activities and CSR reporting. Conceptually, there are arguments for both a positive and negative relation (Hummel and Schlick 2016). Disclosure theory suggests that better performers have incentives to report their performance to stakeholders (and worse performers to hide their poor CSR outcomes). Socio-political theories suggest that poor CSR performers have an incentive to provide positive disclosures to address the threats to their legitimacy from the underlying poor CSR performance (“greenwashing”). Not surprisingly in light of these conflicting predictions, the empirical evidence on the link between CSR reporting and performance is decidedly mixed (see also Section 4.1). For instance, examining the relation between environmental performance and the respective CSR disclosures, Cho and Patten (2007) find a negative association, whereas Clarkson et al. (2008) find a positive one. Other explanations for the mixed evidence include confounding or omitted factors, sample selection issues, and measurement problems related to the underlying CSR performance (e.g., Patten 2002).

To address issues like these, Huang and Lu (2021) exploit a U.K. setting where gender pay gap disclosures became mandatory in 2017. They presume that mandatory disclosures are subject to less measurement and selections issues and use them to evaluate the voluntary pay gap disclosures in 2015. They find that the firms with the lowest gender pay gaps in 2017 voluntarily disclosed the least in 2015, and that firms with larger gender pay gaps had higher ESG social scores.

Another strand of literature focuses on the timing of CSR reporting. Studies attempt to explain when firms initiate CSR reporting (e.g., Bebbington et al. 2009; Belal and Owen 2015) or why some firms adopt CSR reporting early whereas others wait until later (e.g., Kolk 2010; Höllerer 2013; Stubbs and Higgins 2014; Luo et al. 2017). The evidence suggests that following events such as natural catastrophes or environmental accidents, firms increase their CSR disclosures. For instance, Patten (1992) finds that after the Exxon Valdez oil spill in 1989, there was a significant increase in CSR disclosures by petroleum firms other than Exxon. A similar effect is documented around the BP oil spill in 2010 (Heflin and Wallace 2017) and the Fukushima nuclear disaster in 2011 (Bonetti et al. 2018). These event-type studies allow for difference-in-differences specifications in which the treatment is arguably independent of firms’ reporting incentives. However, the variety of settings illustrates that the timing and content of CSR disclosures are often quite idiosyncratic, resulting in heterogeneous reporting practices.

### External stakeholder and societal pressure

There is evidence that external pressure from stakeholders affects CSR reporting (Huang and Watson 2015). Shareholders are a likely source of this pressure and, hence, can be important in shaping CSR reporting (e.g., Gamerschlag et al. 2011). For instance, Reid and Toffel (2009) find that shareholder resolutions filed by social activists can increase the propensity to disclose CSR information both for the firm for which the resolution is filed and for other firms in the industry. Institutional investors constitute a particularly powerful group because of the capital stake they control and their level of specialization and sophistication, as compared with retail investors. Several papers suggest that institutional investors play a role in pressuring firms to initiate and consequently adjust CSR reporting to better reflect the preferences of their institutional owners (e.g., Dhaliwal et al. 2011; Solomon et al. 2011; Pawliczek et al. 2020). Relatedly, there is evidence that CSR activities are affected by institutional ownership (Chen et al. 2020; Dyck et al. 2019) and that investor sentiment towards CSR varies over time (Naughton et al. 2018).

Governments and policymakers represent another important stakeholder group. Aside from their direct influence through mandates or regulation, they can indirectly affect firms’ reporting. Political considerations and the scrutiny by public authorities often motivate voluntary CSR activities (e.g., Doonan et al. 2005; Delmas and Toffel 2008; Innes and Sam 2008) and lead to CSR reporting along the lines of “do good and talk about it.” For instance, Reid and Toffel (2009) provide evidence that the threat of future government regulation can motivate firms to initiate or extend CSR reporting. Similarly, Marquis and Qian (2014) show that government dependence—due to state ownership, political ties, or subsidies—can induce firms to issue CSR reports if they perceive that the reports and activities receive political attention. The study also suggests that greater government monitoring leads to more substantive reports. Some authors think of CSR activities as a form of political contribution (Liston-Heyes and Ceton 2007) and a means to legitimize corporate actions towards regulators. Similarly, firms could provide CSR information to legitimize their actions towards consumers, employees, nongovernmental organizations (NGOs), and politicians or to convey that they are acting in the broader interests of society (e.g., Deegan 2002; Cho and Patten 2007; Deegan 2007; Cho et al. 2015b). Along similar lines, Aerts and Cormier (2009) find that firms’ environmental disclosures and press releases shape media coverage on the topic. But their findings also suggest a reverse effect, in that negative media coverage can induce firms to issue (more) environmental press releases.

### Implications for mandatory adoption of CSR standards

The aforementioned findings have a number of implications for a CSR reporting mandate. First, descriptive evidence in the literature proposes a large set of determinants of voluntary CSR reporting. Yet, the observed associations suggest that there is significant overlap in the economic forces that drive voluntary CSR reporting and the economic drivers of voluntary disclosures in general. It is, in part, this commonality that makes it difficult for researchers to separately estimate the effect of CSR disclosures on capital market (and other) outcomes, because these outcomes likely also reflect firms’ other, non-CSR disclosure choices. However, there are also determinants that are specific to CSR reporting (e.g., a response to an environmental catastrophe, the gender of CEOs’ children, or investor sentiment towards CSR).

Second, there exists considerable heterogeneity in what and how firms report about their CSR activities. An analysis of CSR reporting practices in regulatory filings shows that a majority of U.S. public firms disclose at least some CSR information.^19^ However, the disclosures are often repetitive (within the same report) and, in many cases, are boilerplate and not tailored to the reporting firm. The disclosure of quantitative CSR metrics is still rare in regulatory filings (SASB 2017c) but relatively common in stand-alone CSR reports (Li et al. 2021). The disclosure levels also vary greatly across sectors and firm size. Large firms and those operating in regulated industries with much governmental influence tend to cover more CSR topics. These firms are also more likely to disclose specific narratives and quantitative metrics. The heterogeneity in reported CSR topics makes it difficult for users to compare disclosures and to benchmark firms’ underlying CSR performance. We note that the observed lack of harmonization is not necessarily problematic. High variation is, at least in part, explained by heterogeneity in firms’ business activities and in the materiality of these activities as well as in firms’ perceived costs and benefits of CSR disclosures.

Third, the presence of substantial heterogeneity in CSR reporting practices suggests that a CSR reporting mandate and the use of CSR standards have the potential to increase the level and specificity of firms’ disclosures. To the extent that compliance with such CSR reporting standards is well enforced, the increase in disclosures relative to what firms currently report is likely greater for small firms and in less regulated sectors or those with little government interference. However, low current disclosure levels could also reflect revealed preferences (e.g., higher costs or lower benefits). Thus, past (voluntary) reporting practices predict that compliance issues, if CSR standards were mandatory, would likely be more severe for such firms and industries (in line with the reporting incentives argument in Section 2.4.3).

Fourth, in light of the current heterogeneity in CSR reporting, mandatory CSR standards could increase the level of reporting harmonization, primarily within industries. Such harmonization, in turn, can increase users’ ability to compare CSR information across firms within the same industry, leading to capital market consequences and real effects (see Sections 4 and 5). The comparability benefits would not only accrue to firms with currently low levels of CSR disclosure, but would also lead to better comparisons for best-practice firms. But again, it is important to recognize why current practices are heterogeneous in the first place. Comparability benefits critically hinge on firms’ reporting incentives and their ability to avoid disclosure through boilerplate language or by claiming the information is immaterial, as well as on the enforcement of CSR standards (see Section 6).

Finally, we acknowledge that current CSR reporting and any mandate of CSR standards must be seen in the context of a longer trend towards more and more standardized CSR disclosures. There has been a steady increase in the number of firms that disclose CSR information over the last 25 years (e.g., Serafeim and Grewal 2016; Stolowy and Paugam 2018). What began as voluntary CSR disclosures by firms thrown into the public spotlight after high-profile disasters or scandals (e.g., the Exxon Valdez oil spill) has evolved into industry best practices or guidelines and, in some countries, culminated in CSR disclosure mandates (e.g., in the EU, South Africa, or China). Moreover, CSR activities that were once seen as not value enhancing have become value enhancing over time, as stakeholder preferences and themes in the public debate changed. Thus, the evolution of CSR reporting indicates a surge in market demand for CSR information and an increasingly positive cost-benefit tradeoff for firms over time. It also suggests that CSR has a time-variant component, which can make the extrapolation of prior empirical evidence into the future difficult. These trends will likely continue in the years to come and result in more (but likely also different) CSR reporting even in the absence of a mandate.

## Potential stakeholder effects of mandatory CSR reporting standards

In this section, we discuss potential effects of a CSR reporting mandate with respect to various users of CSR disclosures. We separately discuss each user group, such as investors or consumers, although in practice a given individual may belong to several groups. We begin by briefly reviewing the link between CSR activities and firm value or performance (Section 4.1). This link is central to the CSR literature and is an important motivation for investors to gather information about CSR. We then discuss potential capital-market effects, focusing on equity investors (Section 4.2) and debtholders or lenders (Section 4.3). Next, we turn to financial and information intermediaries, such as analysts and the business press, that play a role in the dissemination of CSR information (Section 4.4). Finally, we analyze potential effects on stakeholders without a direct financial claim on the firm, such as customers, employees, or the society at large (Section 4.5). We conclude with a discussion of the implications of the various stakeholder effects for a CSR reporting mandate (Section 4.6).

### Link between CSR activities and firm value and performance

A critical element of the CSR debate is the question of how CSR activities relate to firm value or financial performance (e.g., Mackey et al. 2007; Kitzmueller and Shimshack 2012). The traditional starting point in this debate is the notion that managers should only engage in activities that increase or maximize shareholder value (Friedman 1962).^20^ Obviously, CSR activities with positive NPV are compatible with this objective (e.g., investments in eco-friendly technologies that save costs, avoid fines, or allow firms to set higher prices in the marketplace). Such activities are not much different from “regular” corporate investments with positive NPV. However, firms could also pursue CSR activities with negative NPV and still act in the interest of shareholders, when (at least some) shareholders put a non-monetary value on CSR or have preferences for specific CSR issues (e.g., Fama and French 2007; Friedman and Heinle 2016; Hart and Zingales 2017). Here, the firm maximizes shareholder welfare but not shareholder value (Hart and Zingales 2017). Firms can also engage in CSR because it is in the interest of stakeholders other than investors (e.g., sponsoring of community events, engagement in corporate philanthropy). In addition, managers may use CSR to pursue personal goals or for their private benefit, in which case CSR gives rise to an agency problem (e.g., Masulis and Reza 2015).^21^

Given the broad range of motives for CSR, it is perhaps not surprising that empirical studies examining the relation between CSR and firm value find mixed results (see Table A3, Panel A, in the Online Appendix). That is, studies find significant associations between firm value and various measures for CSR activities or policies, but there is no consensus on the sign of the association. In estimating the relation, many studies face major selection problems because of the voluntary nature of CSR. Consistent with selection, we often see a positive association between *voluntary* CSR activities and firm value, whereas Manchiraju and Rajgopal (2017) find that forcing firms in India to spend 2% of income on CSR has negative valuation effects. In addition, the literature suggests a number of mediating factors that can alter the sign and the strength of the relation between CSR and firm value. Examples are customer satisfaction through better product quality and the ability to innovate (Luo and Bhattacharya 2006), customer awareness of CSR (Servaes and Tamayo 2013), support from external stakeholders (Henisz et al. 2014), investors’ affective reactions to CSR performance (Elliott et al. 2014), and positive media coverage (Cahan et al. 2015). These examples illustrate that at least some CSR activities could well be in the long-run interest of shareholders. The discussion also hints at yet another channel through which CSR activities and ESG exposures could directly affect firm value: firm risk. We discuss this channel in Section 4.2.3.

A different way to examine the potentially value-enhancing effects of CSR is to study its association with financial performance (e.g., return on assets), essentially testing whether companies “do well by doing good.” A large number of studies broadly examine this relation, and their results are again mixed (see Table A5 in the Online Appendix). Many studies show a positive association between CSR and firm performance (e.g., Herremans et al. 1993; Simpson and Kohers 2002; Flammer 2015; Cornett et al. 2016), but there is also evidence that the relation can be U-shaped (e.g., Brammer and Millington 2008). Kitzmueller and Shimshack (2012) conclude in their literature review that extant studies do not provide strong support for CSR having a positive effect on firm profitability, pointing to, among others, Margolis et al. (2009), who perform a meta-analysis of 251 studies. The latter finds a positive correlation between CSR performance and financial performance, but it is small in economic magnitude. Several other meta-studies find a more robust positive relation between CSR performance or activities and financial performance (e.g., Orlitzky et al. 2003; Busch and Friede 2018; Atz et al. 2020). One difficulty in aggregating results across studies is that they often examine different aspects of CSR performance. Thus, the heterogeneity in results can also reflect that the CSR-performance relation differs across the dimensions of CSR (Atz et al. 2020). Moreover, several studies suggest that the relation between CSR and financial performance depends on mediating factors, namely, firm-level innovation and industry-level differentiation (Hull and Rothenberg 2008), a firm’s intangible resources (Surroca et al. 2010), reputation and competitive advantage (Saeidi et al. 2015; Busch and Friede 2018), and how a firm implements its CSR strategy (Tang et al. 2012).

Much of the evidence is not necessarily causal, and is based on largely voluntary firm choices. The relation could therefore run the other way, from financial performance to CSR, in the sense that firms that do well are more likely to engage in CSR (e.g., Waddock and Graves 1997; Margolis et al. 2009). Combining studies in a meta-analysis does not address this underlying selection problem in CSR activities.

### Equity investors as recipients of CSR reporting

The potential link between CSR activities and firm value or performance makes clear that equity investors should care about CSR information. We review the CSR-specific literature with respect to equity-market effects (see Table A3 in the Online Appendix). Specifically, we discuss potential effects of CSR reporting on firm value (Section 4.2.1), stock returns, and market liquidity (Section 4.2.2), as well as on firm risk and cost of capital (Section 4.2.3). Finally, we analyze the effects of CSR reporting on investors’ asset allocation and portfolio holdings, which, in turn, can convey investors’ preferences for certain CSR issues to firms (Section 4.2.4).

#### Effects of CSR reporting on firm value

There is only scant evidence on the direct effects of CSR *reporting* on firm value and firm performance. Plumlee et al. (2015) find that the quality of voluntary CSR disclosures and firm value are positively associated, both through cash flow and discount rate components. Yet, Cho et al. (2015b) find no such relation in their valuation tests. Gao and Zhang (2015) argue that socially responsible firms are less likely to smooth earnings so that they deviate from long-term, permanent earnings. As a result, earnings should be more value relevant, which in turn could increase firm value. Consistent with this idea, Gao and Zhang (2015) find a positive relation between Tobin’s q and the interaction between CSR scores and earnings smoothness.

A key concern about all these findings is that whatever causes firms to voluntarily engage in CSR and then to report those activities also increases financial performance or firm value. Thus, the association with CSR reporting could be spurious. For instance, both the decision to engage in CSR activities and to report about them could be driven by future growth opportunities (e.g., Lys et al. 2015). Since growth opportunities are often unobservable, it is difficult for researchers to isolate their effects on firm value.^22^ Stakeholder preferences and firms’ motivations for voluntary CSR reporting could matter as well (e.g., whether firms act as good corporate citizens or want to be transparent about their ESG activities; see Boesso et al. 2013). Moreover, firms with CSR reporting could differ in their fundamentals from firms without such reports (see Section 3). Put differently, CSR reporting is often endogenous in two ways: it depends on (i) firms’ voluntary CSR activities and (ii) firms’ choices in reporting about these activities. This dual endogeneity makes it hard to identify the valuation effects of CSR reporting.

One way to mitigate selection is to study CSR disclosure mandates. We discuss two studies on equity market effects here. Ioannou and Serafeim (2017) compare firms from four countries with CSR disclosure mandates before 2011 (i.e., China, Denmark, Malaysia, and South Africa) to propensity-matched benchmark firms in a difference-in-differences design. They find that treated firms significantly increase the volume and quality of CSR disclosures after the mandate and are more likely to (voluntarily) seek assurance for these disclosures or to adopt reporting guidelines. The increases in CSR disclosures occur even though the mandates contain “comply or explain” clauses, which would allow firms to opt out of the additional disclosures at relatively low cost. The authors interpret the results as a “race to the top.” They also show that increases in CSR disclosure are associated with higher Tobin’s q.^23^ However, it is difficult to disentangle the reporting effects from the effects of potential changes in the underlying CSR (or any other firm) activities around the reporting mandate. For this reason, Ioannou and Serafeim (2017) also use the CSR mandate as an instrument for the observed disclosure changes. Such an instrumental-variable design requires that the valuation effects of the mandate only go through this channel. This exclusion restriction is difficult to satisfy and would be violated if, for instance, the economic circumstances that gave rise to the mandate also affect firm value directly.

Chen et al. (2017) exploit the mandate of two Chinese exchanges that requires mostly larger firms to provide a CSR report. Treated firms have to report on a broad set of topics, including consumer protection, environmental issues, and social welfare services. The study finds that firms that are subject to the CSR reporting mandate experience a reduction in future profitability (but an improvement in environmental outputs). The drop in performance after the mandate again illustrates that the selection issues in settings of voluntary CSR disclosure (which typically show positive valuation or performance effects) could be quite severe.

#### Effects of CSR reporting on stock returns and market liquidity

Event studies using short-window stock returns are a simple way to examine whether CSR disclosures are informative to shareholders. In such event studies, it is easier to attribute market reactions to the CSR disclosures. In contrast, long-window returns or value relevance studies tell us that CSR disclosures and stock returns reflect common information, but do not imply that shareholders learned this information from the CSR disclosures (or responded to them; e.g., Holthausen and Watts 2001). The difficulty of short-window event studies is to separate the news content of the disclosures (about CSR activities) from the valuation effects of CSR reporting per se. To identify reporting effects, it is better to conduct long-window studies around exogenous changes in CSR reporting, but such changes are rare and often limited in scope.

There are numerous studies showing that stock markets respond to the release of negative or positive CSR news, often in the same direction of the news (e.g., Flammer 2013). However, in quite a few cases, market reactions and CSR news go in opposite directions, suggesting that shareholders and other stakeholders do not always agree in their interpretations (e.g., events with positive impact on the environment are accompanied by negative market reactions; Groening and Kanuri 2013).^24^ Krüger (2015) finds that investors respond negatively to bad CSR news and have weakly negative reactions to good news. He also shows that the price reaction depends on whether investors perceive an event to be indicative of agency conflicts with management. Several studies suggest that the market reactions to CSR news are asymmetric, with investors putting more weight on negative news (e.g., Flammer 2013; Crifo et al. 2015). Christensen et al. (2017) examine whether market reactions are stronger when CSR events are also reported in 8-K filings with the SEC and not just posted on a government website. The authors find stronger market reactions when certain mine-safety citations are included in both outlets, suggesting that inclusion in SEC filings aids information dissemination and price formation.

A related and recurring theme in the literature is that CSR activities and CSR reporting offer a form of ex ante “insurance” in case something subsequently goes wrong. This insurance effect of a firm’s CSR reputation has been shown for CSR activities that create goodwill with outside stakeholders or society at large (Godfrey et al. 2009; Hoepner et al. 2019); around corporate scandals and high-profile misconduct (Janney and Gove 2011; Christensen 2016); after inadvertent (non-fraudulent) financial restatements (Bartov et al. 2020); following negative press coverage (Shiu and Yang 2017); and during adverse macroeconomic shocks such as the 2008 financial crisis (Lins et al. 2017), the 2010 BP oil spill in the Gulf of Mexico (Heflin and Wallace 2017), or the Covid-19 market crash (Albuquerque et al. 2020).^25^ However, the same studies also suggest that a well-established CSR reputation can backfire or have negative effects, as, for instance, in cases of repeated occurrences of negative (CSR) events, of outright fraud, of delayed CSR disclosures, or if the events occur in a CSR area that was previously touted as important.

For the most part, the studies above speak to how the market reacts to new information about the underlying CSR activities and exposures rather than to CSR reporting per se. Highlighting this distinction, Jouvenot and Krueger (2020) find that return reactions to first-time carbon disclosures following the U.K. disclosure mandate are more negative for firms that reveal larger emissions. Focusing on the reporting dimension, Grewal et al. (2020) find a negative association between a disclosure score for material CSR information and stock price synchronicity. They interpret the results as consistent with only select CSR disclosures being relevant to investors and increasing the amount of firm-specific information incorporated into price. Becchetti et al. (2015) provide evidence that CSR scores and idiosyncratic volatility (a proxy for firm-specific information) are positively related. They argue that CSR reduces the ability of firms to respond to negative productivity shocks, essentially putting a constraint on firm behavior. However, it is again not entirely clear how the design is able to separate CSR reporting from the underlying CSR activities.

One of the few studies to analyze the announcement returns to a CSR reporting mandate is Grewal et al. (2019). The authors examine (short-window) returns to events leading up to the passage of an EU Directive mandating the disclosure of nonfinancial (CSR) information by firms. The study finds, on average, a negative market reaction, but less negative or even positive returns for firms with more pre-directive CSR disclosures and stronger CSR performance. The results suggest that investors view the CSR reporting mandate as costly, particularly for firms that provide few voluntary CSR disclosures and would be forced to disclose additional CSR information.^26^ The study also shows that the negative reaction is related to proprietary and political costs. Hombach and Sellhorn (2018) find similar results around the passage of an SEC rule mandating project-level disclosures of payments made to governments by extractive issuers. The market reactions are more negative for firms subject to greater public scrutiny, consistent with investors expecting costly changes to firms’ business activities. The latter result highlights one of the significant challenges of regulatory event studies. The market reaction not only reflects the capital-market effects of CSR reporting but also potential real effects that follow from the reporting mandate. Other empirical challenges include the difficulty in cleanly identifying the dates on which investor expectations change and how anticipation of future regulation plays into the results (e.g., Binder 1985; Leuz 2007), as well as the fact that the typical regulatory event study uses a limited number of events that are common to all sample firms and, hence, prone to concerns about confounding events and concurrent industry- or economy-wide shocks.

There is relatively limited evidence on the liquidity consequences of CSR reporting, which is perhaps surprising considering that liquidity tests are well suited to isolate information effects in markets. In this vein, Barth et al. (2017) show that following the 2010 integrated reporting mandate for companies listed on the Johannesburg Stock Exchange, firms with higher-quality integrated reports and with larger yearly changes in reporting quality have lower bid-ask spreads (and higher firm value).^27^ Grewal et al. (2020) find a negative relation between material CSR disclosures and bid-ask spreads or zero return days. Cho et al. (2013) find a negative association between CSR performance scores and information asymmetry. This relation holds for both positive and negative CSR performance. Using a set of Canadian firms, Cormier and Magnan (1999) show that trading volume is positively associated with a voluntary CSR disclosure score.^28^

#### Effects of CSR reporting on firm risk and cost of equity capital

Broadly speaking, there are two ways in which CSR activities and exposures can manifest in the cost of the capital. One way is that certain business activities pose CSR-related risks (e.g., fossil fuel producers face risks from the transition to carbon neutrality). To the extent that these risk exposures are not diversifiable for investors, they manifest in firms’ cost of capital. That is, firms that are less susceptible to CSR or ESG shocks offer lower returns, and vice versa. Engaging in certain CSR activities could mitigate these risks or risk exposures. One argument being put forth is that CSR generates “moral capital,” for instance, through customer trust, employee loyalty, lower price elasticities, or goodwill with regulators (e.g., Luo and Bhattacharya 2009). Such moral capital provides insurance-like protection against negative future events and the reactions of stakeholders to such events, resulting in a lower volatility of future cash flows.

The other way to relate CSR activities to the cost of capital is via investor preferences. Fama and French (2007) and Friedman and Heinle (2016) show that if some investors have nonfinancial CSR preferences, they are willing to accept lower expected returns from firms that satisfy these preferences, resulting in a lower cost of capital. Pástor et al. (2020) reaffirm this conclusion and further show that if ESG preferences shift over time, they can become a non-diversifiable risk factor and firms’ exposures against this factor are priced in the cost of capital.

Illustrating that CSR activities are related to firm risk and the cost of capital, studies find that better CSR performance is negatively associated with idiosyncratic risk (Luo and Bhattacharya 2009),^29^ crash risk measured as the negative skewness of stock returns (Kim et al. 2014b), the risk of future stakeholder conflicts (Becchetti et al. 2015), and systematic risk using beta estimates (Albuquerque et al. 2018). Studying carbon risk exposures, Bolton and Kacpercyk (2020a, 2021) find that firms with higher carbon emissions have higher equity returns, consistent with a carbon premium, both for U.S. companies and globally.

However, it is important to recognize that the aforementioned links to the cost of capital—whether conceptual or empirical—are for the underlying CSR activities and exposures, not for CSR reporting. For the latter, we expect CSR disclosures to behave similarly to other firm disclosures. That is, better information should lower firms’ cost of capital to the extent that (i) it reduces information asymmetry and improves liquidity and, hence, transaction costs to investors (Amihud and Mendelson 1986), or (ii) it reduces the conditional covariances that investors use to compute the factor betas (Lambert et al. 2007).

Studies focusing on reporting provide evidence of a negative relation between voluntary CSR disclosures and firms’ implied cost of capital (e.g., El Ghoul et al. 2011; Plumlee et al. 2015; Matsumura et al. 2017) and stock returns (Bolton and Kacpercyk 2020b). The relation often depends on a number of mediating factors, such as firms’ actual CSR performance (Dhaliwal et al. 2011), the type of CSR disclosures (e.g., environmental and governance topics; Ng and Rezaee 2015), whether the CSR disclosures are material to investors (Matsumura et al. 2017), or whether a third-party provides assurance of the CSR reports (Casey and Grenier 2015). Other studies find no relation between CSR disclosures and cost of capital (Clarkson et al. 2013), or even a positive relation (Richardson and Welker 2001).

But as noted before, it is very difficult to differentiate between CSR activities and reporting. Many of the above studies face the issue that firms with voluntary CSR disclosures could differ systematically in their CSR activities and, hence, firm risk or cost of capital. To disentangle the two, Bonetti et al. (2018) analyze the cost of capital effects of firms’ environmental disclosures around an arguably exogenous shock (i.e., the Fukushima nuclear disaster in 2011). They find evidence that Japanese firms with stand-alone environmental reports incur a less severe cost-of-capital increase after the disaster than firms without such a report. There is a smaller market response for firms with more credible CSR disclosures. In additional tests, the authors find that firms increase their voluntary CSR disclosures in the aftermath of the disaster.

#### Investor preferences and effects of CSR reporting on portfolio holdings

One reason to analyze effects on investor portfolio holdings is that investors can have different tastes for CSR activities (e.g., Fama and French 2007; Friedman and Heinle 2016). These tastes give rise to investor clientele or shareholder base effects, which can feed back into firms’ (CSR) activities.^30^ Examples of feedback effects are shareholder proposals on CSR issues by activist investors that subsequently are related to better CSR performance (Grewal et al. 2017). Dimson et al. (2015) find that successful CSR campaigns by active owners are associated with positive stock returns and better accounting performance. These findings suggest that investor preferences and actions by institutional investors could be an important mechanism for real effects from CSR reporting (see Section 5).

Experimental research by Martin and Moser (2016) finds that investors respond favorably to CSR disclosures highlighting societal benefits, even when the underlying activities are net costly. Similarly, El Ghoul and Karoui (2017) find that investors in mutual funds with portfolios of high-CSR-performing firms appear to derive utility from non-financial-performance attributes. Hartzmark and Sussman (2019) exploit the introduction of CSR ratings by Morningstar and show that perceptions about sustainability drive mutual fund flows and that investors place a positive value on CSR ratings. Bolton and Kacpercyk (2021) find that institutional investors implement exclusionary screening based on CO_2_ emissions in industries in which emissions are salient (e.g., oil and gas). More nuanced findings suggest that professional investors focus primarily on CSR information that they consider financially material (Amel-Zadeh and Serafeim 2018), that individual investors perceive CSR disclosures as more important (and as increasing their willingness to invest) if CSR activities are relevant to the firm’s strategy (Cheng et al. 2015), and that CSR disclosures affect investor judgments more when presented in stand-alone reports (Bucaro et al. 2020). Finally, there is evidence that investors with long-term horizons are more likely to invest in firms with strong CSR performance or behave more patiently towards these firms by selling less after negative earnings surprises and poor returns (e.g., Gibson et al. 2020; Starks et al. 2017). These findings suggest a link between investment horizon and CSR preferences.

### Lenders and debtholders as recipients of CSR reporting

Similar to equity investors, lenders can have a taste for CSR (e.g., Barigozzi and Tedeschi 2015). For instance, closer matching of interests between firms and their lenders could reduce inherent agency conflicts, compensating for the higher costs of CSR activities.^31^ Such borrower-lender matching can have credit market effects. Hasan et al. (2017) find that firms headquartered in communities with higher social capital exhibit lower loan spreads. Hauptmann (2017) shows that banks with strong CSR performance provide more favorable loan spreads to firms with equally strong CSR performance. In addition, several studies find that better CSR performance is associated with lower loan spreads and, hence, a lower cost of debt (Goss and Roberts 2011; Chava 2014; Cheng et al. 2014; Kim et al. 2014a; Kleimeier and Viehs 2018; Cheng et al. 2017).^32^

Related to these studies, several papers use the municipal-bond market to examine the premium of green bonds over traditional bonds. Baker et al. (2018) compare across green and traditional bonds and find that green bonds are associated with lower yields, whereas Larcker and Watts (2020) compare green and traditional bonds issued on the same day and find no evidence of a premium for green bonds. Potentially explaining these apparently conflicting findings, Lu (2021) argues and shows that green bonds can act as a device to commit to CSR activities or green investments, and that bond markets reward such commitment with lower cost of debt for both green and traditional bonds issued by the same municipality.

Similar to the insurance effects in equity markets, the negative association between CSR and loan spreads is more pronounced in periods of low trust and crisis, such as during the 2008-2009 financial crisis (Amiraslani et al. 2021). CSR bonds appear to outperform conventional bonds during down markets (Henke 2016). Evidence that CSR disclosures can limit the impact of negative events and damaging revelations about a firm should be relevant for debtholders, as they naturally are more interested in the downside risk of their holdings. Conversely, credit markets are shown to react negatively when a firm displays or the press uncovers irresponsible CSR behavior and the firm is involved in CSR scandals (Kölbel et al. 2017). Finally, CSR performance is negatively related to capital constraints, with CSR disclosures playing a moderating role in this association (Cheng et al. 2014).

Overall, the literature on debt-market effects is primarily focused on firms’ CSR activities (see Panel A of Table A4 in the Online Appendix). There is not much evidence on the effects of CSR *reporting*. A CSR reporting mandate would be relevant primarily for public debt offerings and publicly traded debt, and less so for private placements or bank debt. A mandate likely would reduce the search and information processing costs for bond investors and should make it easier to compare firms, particularly if investors have preferences for certain CSR topics. However, as Lu (2021) shows, there are also ways for firms to privately create commitments for extended CSR activities and CSR reporting.

### Analysts and the media as recipients of CSR reporting

Financial intermediaries like analysts and the media also take an interest in firms’ CSR. The issuance of a stand-alone CSR report and certain disclosures indicating CSR strengths are negatively associated with analyst forecast errors (e.g., Dhaliwal et al. 2012; Becchetti et al. 2013). In contrast, CSR weaknesses exhibit a positive association. Analysts appear to put more weight on CSR reports when an external accounting firm provides assurance (Pflugrath et al. 2011). Hope et al. (2016) find that the more specific a firm’s disclosures (including CSR disclosures), the better analysts are able to assess fundamental firm risk. However, it seems that these relations need time to develop. Ioannou and Serafeim (2015) show that analysts’ stock recommendations initially suggest a negative view of firms’ CSR investments, consistent with these activities being costly or a reflection of agency problems. As a firm’s CSR reputation grows, stock recommendations become more favorable.

The media naturally plays a dissemination role for firms’ CSR disclosures. However, there is also evidence that CSR can shape media coverage. Firms with a stronger CSR reputation tend to receive more favorable media coverage, which provides managers with incentives to use CSR as a tool to manage the public image of the firm (Cahan et al. 2015). For instance, firms in the so-called “sin” industries (alcohol, gambling, and tobacco) attempt to actively portray a positive CSR image and reputation. On the other hand, the media reinforces the negative repercussions from poor CSR behavior (Kölbel et al. 2017). Firms that exhibit irresponsible CSR behavior tend to receive a lot of (negative) press attention. Thus, the media are likely an important channel through which disclosures about firms’ CSR activities could have real effects.

A CSR reporting mandate should affect financial analysts and the media in similar ways as equity investors, meaning it should lower search and information processing costs. If CSR disclosures and analyst coverage act as substitutes, standardized disclosures could reduce analysts’ incentives to gather private information about firms’ CSR activities (e.g., Barron et al. 2002). If they are complements, CSR standards could enable analysts to better integrate CSR information into their fundamental analysis and valuation models. Analysts may give more room to CSR issues in their research reports (e.g., PRI Principles for Responsible Investment (PRI) Association 2013). Given that such research reports generally focus on financial performance and valuation, analysts likely have the greatest interest in CSR disclosures that are informative about firms’ future expected cash flows and risks.

The media likely also have a broad interest in CSR. A CSR reporting mandate could make it easier for journalists to compare and rank firms’ CSR strategies. It could also reduce the initial information gathering costs for CSR-related news stories. Consistent with this argument, Christensen et al. (2017) provide descriptive evidence that the inclusion of mine-safety information in regulatory filings increases the use of this information by both analysts and journalists. They find that financial institutions such as brokerage houses and investment banks account for about 50% of mine-safety related 8-K downloads, and the news media accounts for about 26%. The authors also find a substantial increase in media coverage of certain mine-safety citations and a (modest) increase in the frequency with which safety is discussed during earnings conference calls.

### Customers, employees, and other stakeholders as recipients of CSR reporting

Several other stakeholder groups are potential recipients of CSR information (see Panels C to E of Table A4 in the Online Appendix). Specifically, we discuss the role of CSR reporting for society in general, including activists, policymakers and regulators (Section 4.5.1), for customers and consumers (Section 4.5.2), and for employees and management (Section 4.5.3).^33^

#### Society in general

From a societal perspective, CSR and sustainability are about externalities and the distribution of rights and assets across generations (e.g., Howarth and Norgaard 1992). Society can pressure firms to pursue specific CSR goals and behaviors, which often are costly and have no obvious payoffs to shareholders. Interest groups may pressure firms to pursue CSR activities on their behalf, essentially as delegated philanthropists (e.g., Bénabou and Tirole 2010). In this context, CSR reporting (and, of course, the underlying CSR activities) can be used to proactively abate societal pressure (e.g., Reid and Toffel 2009), reduce the risk of harmful regulatory actions (Hillman and Keim 2001), promote corporate goodwill, explain the sustainability of firm strategies, and pander to special interest groups (e.g., activists, customers).

Again, much of the research in this area focuses on the effects of CSR activities rather than reporting. Murray and Vogel (1997) find that CSR has a positive, but often intangible impact on the perceptions of (potential) stakeholders, both at the individual and group level. Stakeholders’ preconceived attitudes toward (or taste for) particular CSR issues are likely to mediate this relation (e.g., Shafer and Simmons 2008). Firms may use CSR strategically to establish a bond with society or to improve their relationships with specific interest groups in return for reputational goodwill and monetary benefits. For instance, Lin et al. (2015) find that Chinese firms expand their spending for politically sensitive CSR topics to curry favor with local politicians and reap benefits in the form of higher government subsidies.

A CSR reporting mandate could have a number of effects. For CSR to be impactful, the various stakeholders need to be aware of it. Voluntary CSR disclosures are one way that firms can convey their CSR activities to the public. However, these disclosures are not necessarily credible, preventing firms from reaping the full benefits. A reporting mandate could provide credible commitment, especially if the standards are well enforced. The type of interaction between the firm and its stakeholders could also affect the usefulness of CSR standards. If interactions are at arm’s length or passive (i.e., stakeholders vote with “their feet” or engage in screening for positive and negative CSR performance), standardized CSR reporting is likely more useful. If interactions are more relational (and friendly), involving a dialogue between the firm and its stakeholders, then CSR information can be customized and privately exchanged, with less need for standardization.

Firms may opt to implement CSR standards in a more “symbolic” way to legitimize corporate actions, selectively disclosing positive CSR activities, without intending to materially adjust the underlying real activities (e.g., O’Sullivan and O’Dwyer 2009; Marquis et al. 2016; Diouf and Boiral 2017).^34^ In doing so, firms can exploit the discretion in standards—for instance, by using boilerplate language to conceal the “true” CSR (in-)actions (Crilly et al. 2016) or by strategically presenting the information in such a way as to influence user perceptions (e.g., Cho et al. 2009). This so-called “greenwashing” aims at hiding negative actions through positive, but merely symbolic, activities and reporting.^35^ As Wu et al. (2020) analytically show, the amount of greenwashing depends on the level of transparency about firms’ CSR activities, and higher transparency can incentivize socially responsible firms to increase their (observable) CSR investments.

#### Customers and consumers

Customers exhibit a variety of preferences and views with respect to CSR, which may or may not map into firms’ CSR activities. If there is fit (e.g., common interest in “green” manufacturing), firms’ CSR engagement likely positively affects consumer perceptions. In turn, customer loyalty could go up, potentially boosting future sales and increasing customers’ willingness to pay for products and services (e.g., Navarro 1988; Eichholtz et al. 2013; Grimmer and Bingham 2013; Park et al. 2014; Habel et al. 2016).^36^ The type of CSR activities, consumers’ personal values, and a firm’s ability to innovate and ensure product quality act as mediating factors for these associations (e.g., Luo and Bhattacharya 2006; Homburg et al. 2013; Öberseder et al. 2013).

Trust by customers can also turn into skepticism. In response to CSR claims that turn out to be unsubstantiated, CSR incidents, or corporate hypocrisy, customers can become critical about a firm’s CSR activities and change their initially positive view (e.g., Wagner et al. 2009; Skarmeas and Leonidou 2013; Skarmeas et al. 2014). Standardized CSR reports might serve as a starting point for consumers, who are typically less informed and sophisticated than investors in assessing a firm’s CSR, and could help them with peer comparisons. At the same time, one-size-fits-all CSR standards can create a disconnect between what firms must report and what customers deem important (e.g., Bradford et al. 2017).

#### Employees and management

CSR has the potential to increase employee loyalty, which could lead to lower wage expenditures, either because employees are willing to work at lower rates or by increasing employee retention and intrinsic motivation (Greening and Turban 2000). In this regard, CSR reporting can play a role in communicating and dispersing the message across the firm. Ways to incentivize employees include tying CSR goals to management compensation (Mio et al. 2015) or integrating CSR goals into the existing management control system (e.g., Collison et al. 2009; Arjaliès and Mundy 2013). CSR reporting could also serve as a disciplining tool for the supply chain (e.g., Spence and Rinaldi 2014; Dai et al. 2020; Darendeli et al. 2021).^37^ Moreover, Gao et al. (2014) find that managers from CSR-conscious firms engage less in self-serving insider trading and, if they do engage, reap lower benefits from these trades. This result is consistent with CSR making insider trading more costly. Alternatively, it could reflect a selection or matching effect. CSR-conscious firms hire managers that are more ethical and less likely to engage in opportunistic trades.

Windolph et al. (2014) find that the response to CSR incentives and the extent to which employees pursue CSR goals vary substantially across functional areas within the firm, such as marketing, sales, and finance. Employees and managers might be influenced by the perceptions of their roles within the organization. For instance, Armstrong (1977) finds that exposing managers to CSR information or asking them to explicitly represent external stakeholder interests improves their socially responsible behavior. Aggregate and multifaceted CSR goals may be difficult to communicate and break down into measurable performance indicators (e.g., Virtanen et al. 2013). For instance, Mäkelä and Näsi (2010) find that managers and employees differ in their interpretation of the same CSR messages. O’Dwyer (2003) suggests that managers feel constrained by the financial performance goals of the firm when pursuing CSR goals. On a more practical level, the main obstacles encountered when implementing CSR reporting for internal purposes are issues like data availability, additivity of measurement units, reliability of estimates, as well as resistance by managers and employees (e.g., Herbohn 2005; Virtanen et al. 2013).

### Implications for mandatory adoption of CSR standards

From the above discussion, it is clear that capital-market participants have a demand for CSR information, not least because of the potential performance, risk or valuation implications. Other stakeholders and society care about CSR information in light of the potential externalities of corporate activities and firms’ broader impact in ESG areas. Voluntary disclosures are one way for firms to convey this information. However, voluntary disclosures are not always credible or effective. Stakeholders might worry that such disclosures are provided only when the information is favorable. Although a CSR reporting mandate could partly overcome these deficiencies, there are also reasons to be skeptical about the ability of standards to force out information that firms do not want to disclose (see also Section 2.4.3). Thus, the effect is ultimately an empirical question.

Empirical evidence on the effects of CSR *reporting* is limited and still developing. While there are many studies that rely on reported CSR information, only a few focus on the reporting effects per se, and those that do face the challenge of disentangling the effects of CSR reporting from the underlying CSR or business activities. Moreover, most studies rely on voluntary CSR disclosures, which are subject to a dual selection problem (see Section 2.3). Even when these studies are able to identify (causal) effects, we typically cannot use their results to justify a mandate. The voluntary nature of firms’ choices makes the observed effects likely not representative for the population of firms (Leuz and Wysocki 2016). Consistent with the presence of selection effects, voluntary CSR disclosure studies often provide evidence of beneficial effects, whereas studies on mandatory CSR reporting find less or no capital-market benefits. One reason for this discrepancy could be that CSR reporting mandates are often designed not with investors but with other stakeholders in mind.

Against this backdrop, we derive the following potential implications of a CSR reporting mandate for the various stakeholder groups. First, to the extent that CSR disclosures provide new or better information, they should have tangible capital-market benefits in the form of improved liquidity, lower cost of capital, and higher asset prices (see Section 2.4.1), especially when the information is material to investors. At the same time, CSR reporting is costly. A key concern is that it could reveal proprietary information to competitors, customers, and suppliers, which could reduce incentives to engage in innovative CSR activities in the first place (Breuer et al. 2020). The heightened transparency and scrutiny of firms’ CSR could also increase the threat of regulatory actions or litigation by shareholders and other parties.^38^ Thus, the net effects for firms in capital markets are not a priori obvious.

Second, the extent to which a CSR reporting mandate induces firms to provide new and better information critically hinges on firms’ reporting incentives. Evidence from voluntary CSR disclosures suggests that reporting incentives differ substantially across industries and firms (Section 3). Imposing CSR standards will have little effect if firms’ underlying reporting incentives do not change *and* firms have the ability to evade reporting by using boilerplate language or claiming the information is immaterial. These arguments point to the need for institutional reform that provides tools to all stakeholders (not just investors) to hold firms accountable for their CSR reporting (Cooper and Owen 2007). Much will depend on the specificity, proper implementation, and enforcement of the standards. Given the heterogenous nature of CSR, substantial discretion in CSR standards is likely necessary for the reporting to be informative. But such discretion can also exacerbate the aforementioned compliance issues.

Third, it is important to consider what existing U.S. securities regulation and SEC rules imply for CSR reporting. These requirements prohibit SEC-registered firms from omitting material information from regulatory filings irrespective of the nature of the information (see also Section 6.2.1). Therefore, the magnitude of the capital-market effects from a CSR reporting mandate depends crucially on the extent to which firms currently withhold material CSR information. If firms are largely in compliance with the existing rules and do not omit information that is considered material, then CSR standards based on single materiality should not produce much new decision-relevant information for investors. In that case, the primary benefits of CSR standards to investors must largely come from standardization. But it is difficult to assess how much material CSR information firms currently withhold and to what extent there is a compliance issue with current regulations. Peters and Romi (2013) use monetary sanctions by the U.S. Environmental Protection Agency as an item that firms must disclose in SEC filings under Item 103 of Regulation S-K, and show a 72% noncompliance rate. Relatedly, using ESG disclosure data in Bloomberg, Grewal et al. (2020) find that, on average, firms provide only about 18% (median: 13%) of the prescribed SASB disclosure items (which serve as the benchmark for financially material CSR disclosures). These statistics point to substantial noncompliance or underreporting of material CSR information. Thus, a CSR reporting mandate could provide more specificity (e.g., define standards that closely relate to firms’ business activities), so that noncompliance can be detected and the rules can be enforced. In that case, a mandate should force out new material CSR information, and capital markets should respond as theory predicts.

Fourth, a mandate and the ensuing standardization of CSR reporting in substance, format, and presentation should make it easier for all stakeholders to find, process, and compare CSR disclosures. These cost savings arise not just for investors but also for other stakeholders and society at large. Of course, it is not necessarily the case that firms report what the various stakeholders would otherwise be collecting or interested in. A mandate could also make it easier for investors to pursue investment strategies that reflect their CSR preferences and for consumers to make CSR-based consumption decisions.^39^

Fifth, CSR standards could make it easier to benchmark firms’ CSR performance over time and across firms. However, ranking firms produces winners and losers, as not all firms can be at the top. Firms are unlikely to be in full control of their CSR performance (and ranking), as outside factors define part of it (e.g., natural catastrophes and accidents). Moreover, the measurement system for CSR performance is likely incomplete and noisy (e.g., injuries are an imperfect proxy of worker safety). In that sense, mandatory CSR reporting could expose firms to additional reputation risks and increase the likelihood of bad publicity, even when firms are not at fault. Such risks could cause firms to engage in costly (real) activities (see Section 5). Of course, CSR-related risks also exist in the absence of CSR reporting. The media or activists often scrutinize firms irrespective of their CSR reporting (Miller 2006), and stakeholders will not automatically assume that firms without CSR reporting have no CSR issues. Thus, the reputation risks from a CSR reporting mandate likely depend on—among other things—the noise in the mandated CSR metrics and the sophistication (and interests) of stakeholders.

Sixth, due the wide-ranging nature of CSR topics across firms (see Section 2.3), CSR standards likely need a focus on industry or business activity. Such standards would facilitate within-industry comparisons but at the same time hamper cross-industry comparability. Thus, despite the arguably large potential for standardization, it is unlikely that mandated CSR reporting can achieve comparability across all firms. Moreover, it may be difficult for stakeholders to assess a firm’s overall CSR performance.^40^ Firms can do well along some dimensions and poorly along others. Industry-specific CSR scores or rankings are attempts to tackle the aggregation problem. However, such aggregated scores are not without problems. For instance, investors might face difficulties in separating material from immaterial CSR information (e.g., Guay et al. 2016; Dyer et al. 2017). Moreover, CSR scores could incentivize firms to focus on particular outputs or metrics, which is counterproductive if the weights in the score do not reflect the importance of the underlying activities for firms’ CSR performance (e.g., Porter and Kramer 2006). In the extreme, firms could engage in score management and greenwashing (e.g., Cho et al. 2015a).

Finally, a mandate of CSR reporting could make it easier for investors to hold managers accountable, especially when it comes to negative NPV projects. It is not clear that managers have incentives to voluntarily disclose such value-decreasing CSR activities. A credible mechanism that forces firms to disclose unfavorable CSR activities and outcomes could better align managers and shareholders, but might also make it harder for managers to mitigate negative externalities. However, mandated CSR reporting also improves monitoring by stakeholders other than investors, and the resulting pressures could in turn motivate firms to consider such external effects.

## Potential firm responses and real effects from mandatory CSR reporting standards

New CSR disclosures likely not only affect the recipients of the information (see Section 4) but could also induce firms to alter their own behavior, often precisely because they expect investors and other stakeholders to respond to the new disclosures. Thus, this section focuses on the idea of driving change with CSR reporting.^41^ We begin by briefly reviewing the general link between disclosure and firms’ real activities (Section 5.1). We then discuss potential investment, financing, and operating effects of CSR reporting (Section 5.2). Next, we consider the possibility that firms abandon certain activities or exit markets altogether (Section 5.3). We conclude with a discussion of the broad implications of a potential CSR reporting mandate on firms’ real (CSR) activities, both at the individual firm level and for the economy as a whole (Section 5.4).

### General link between disclosure and firms’ real activities

A key insight from extant research in accounting is that disclosure can change a firm’s investment behavior and other real activities (for overviews see Leuz and Wysocki 2016; Roychowdhury et al. 2019). That is, the reporting regime not only informs investors and other stakeholders, but their responses to the disclosures also influence how firms allocate resources (e.g., Kanodia and Sapra 2016). Empirical studies provide examples as well as potential explanations for why corporate disclosures can have real effects, including: (i) disclosure reduces information asymmetry and agency costs, leading to enhanced investment efficiency (e.g., Biddle and Hilary 2006; McNichols and Stubben 2008; Shroff et al. 2014); (ii) accounting treatments (e.g., recognition of stock-based compensation expense) have contractual implications for managerial compensation or debt agreements that induce changes in firms’ investment and spending behavior (e.g., Dukes et al. 1980; Holthausen and Leftwich 1983; Choudhary et al. 2009; Hayes et al. 2012); and (iii) managers learn from peer reporting or use it for benchmarking and subsequently adjust their investments (e.g., Beatty et al. 2013; Chen et al. 2013; Shroff 2017).

To the extent that a CSR reporting mandate forces out new information, it can have real effects on firms’ investments—as can any other corporate disclosure. The disclosure literature suggests that more transparency increases firms’ investment efficiency (i.e., reduces overinvestment and underinvestment; e.g., Biddle and Hilary 2006; Biddle et al. 2009). However, it is unclear how much new information a CSR reporting mandate will produce, which makes it difficult to predict the ensuing (non-CSR) investment responses and efficiency effects. In addition, a CSR reporting mandate likely has effects on firms’ CSR activities. Stakeholder responses to CSR information may induce firms to engage in more CSR and to scale back regular operating investments in favor of CSR investments. But again, it is difficult to predict whether these real effects improve or hurt overall investment efficiency; their net effect on firm value; or the extent to which CSR reporting makes firms internalize externalities.

Another channel through which disclosure can result in real effects is by altering the cost of capital. We see two mechanisms for cost-of-capital-induced real effects (see also Section 4.2.3). First, the cost of capital serves as the hurdle rate for new investments. Thus, when CSR disclosures change firms’ cost of capital, corporate investments should respond accordingly. Several studies point to a negative association between (voluntary) CSR reporting and the cost of capital (e.g., El Ghoul et al. 2011; Plumlee et al. 2015; Matsumura et al. 2017). But there is also evidence that the relation could go the other way (e.g., Richardson and Welker 2001). Note that, if mandated CSR information indeed reduces the cost of capital, firms are expected to increase their investments across the board, not just those related to CSR. Second, firms’ ESG risk exposures as well as investors’ CSR preferences can (directly) affect the cost of capital, which provides incentives for firms to improve their CSR performance, reduce their risk exposures, or align their investments with investors’ CSR preferences (e.g., Pástor et al. 2020). To the extent that CSR disclosures make it easier for investors to assess risk exposures or find firms that match their preferences, CSR reporting could influence firms’ CSR investments through this mechanism.

### Effects of CSR reporting on firm investment, financing, and operating activities

CSR reporting differs from financial reporting in that the potential users and uses of CSR information are much broader. Any user group can use the information even when they are not the primary target audience. As a result, we cannot necessarily extrapolate findings from the financial reporting literature to predict the real effects of CSR disclosures. We first derive potential firm-level implications of a CSR reporting mandate (Section 5.2.1) and then review extant literature on the real effects of CSR reporting (Section 5.2.2).

#### CSR-specific real effects for individual firms

The most likely real effect of a CSR reporting mandate is directly on firms’ CSR activities. In essence, firms are expected to alter their CSR activities whenever (investor and other) stakeholders use the newly disclosed CSR information to exert meaningful pressure on firms (e.g., reduce consumption, withdraw their business, divest their holdings, or instigate activist campaigns). The stakeholder reactions to firm disclosures create a feedback loop in which firms respond to anticipated or actual stakeholder responses. Specifically, the main channels by which standardized CSR disclosures could affect firms’ CSR are: (i) improved monitoring and governance of firms’ CSR activities; (ii) a stronger link between CSR and economic performance; (iii) strengthened market and societal pressure due to newly available CSR information; and (iv) learning about or benchmarking against peer firms’ CSR practices (e.g., Cao et al. 2019).

First, firms may adjust their CSR activities because as more information about these activities becomes available, debt and equity investors are better able to monitor managers’ CSR decisions. The governance channel matters most if some of the current CSR activities are not in the interests of investors and, from that perspective, represent agency conflicts. If a CSR mandate leads to more transparency and allows investors to better assess managerial behavior, they can exert discipline by (the threat of) selling shares or, more directly, through shareholder votes and activism. The result could be a better alignment between the interests of investors and the firm’s CSR activities, leading to a reduction in agency costs and positive effects on firm value.

Second, firms could adjust their CSR activities if they see CSR reporting as having an effect on financial performance. Many studies identify a positive association between voluntary CSR disclosures and financial performance (see Section 4.1). A common justification is that CSR activities build loyalty and trust for the firm and its brands, which can translate into higher future revenues and lower costs (e.g., Cao and Rees 2020). Of course, for CSR to have such an effect, the various stakeholders must be aware it. A CSR reporting mandate, particularly if enforced well, could make CSR information more accurate and credible and easier to process, which would raise stakeholder awareness. If this outcome strengthens the link with financial performance, we expect firms to increase their CSR activities in response (Guo et al. 2017).

Third, higher transparency regarding CSR and the ability of stakeholders other than investors to compare firms against each other at lower costs likely increases societal pressure. In the same way as CSR can build loyalty, poor CSR performance can damage a firm’s reputation and create negative publicity. Stakeholders such as social activists, policymakers, or consumers can exert pressure through actions like public shaming (Dyck et al. 2008), boycotts, or imposing sustainability restrictions along the supply chain (e.g., Dai et al. 2020). In response, firms have incentives to adjust their CSR activities if the (anticipated or perceived) costs from goal misalignment with certain stakeholders are too high.

Finally, better CSR reporting could facilitate inter-firm learning. A CSR reporting mandate would lower the costs of peer benchmarking, especially within the same industry and if the CSR standards focus not just on CSR output metrics but also require information about firms’ CSR activities and processes. In doing so, a CSR reporting mandate could accelerate the adoption of best practices and ultimately improve aggregate CSR performance in the economy (Tomar 2021). The flip side of this argument is that in forcing firms to reveal proprietary CSR information (e.g., low carbon emission technologies), a CSR mandate could reduce firms’ incentives to innovate (e.g., Breuer et al. 2020). Importantly, it is not obvious that the positive spillovers on other firms necessarily outweigh the negative innovation effects.

#### Evidence on CSR-specific real effects

Empirical evidence on the real effects of CSR reporting is still relatively scarce (see Table A6 in the Online Appendix) but is growing fast.^42^ Regulators in many countries (e.g., China, the EU, U.K., U.S., South Africa) have recently imposed CSR reporting mandates on select firms in their jurisdictions.^43^ Studies of these settings are particularly relevant as they provide valuable insights into how firms respond to mandatory CSR disclosures.^44^

In 2008, two Chinese stock exchanges mandated that a subset of their listed firms issue a CSR report together with their annual report. The CSR reports have to cover a broad set of topics, including consumer protection, environmental issues, and the provision of social welfare services. Chen et al. (2017) use this setting to examine how a CSR disclosure mandate affects pollution levels. They find decreases in overall industrial wastewater and SO_2_ emissions in cities with more regulated firms. Consistent with CSR being costly, they also find that firms subject to the disclosure requirements experience a reduction in profitability.^45^

The U.S. also requires select mandatory CSR disclosures. The Dodd-Frank Wall Street Reform and Consumer Protection Act of 2010 includes two: one stipulates that mine owners disclose mine-safety information, and the other requires disclosures related to purchases of minerals from the Democratic Republic of Congo and neighboring countries in firms’ SEC filings. Christensen et al. (2017) examine the real effects of the mine-safety disclosure provisions. The study finds that the safety of mines improves and productivity declines. Thus, the findings line up with those of Chen et al. (2017) for China. One major difference is that, in the U.S. setting, relevant mine-safety information reported in SEC filings was already publicly available on a government website. Thus, the results in Christensen et al. (2017) indicate that including CSR information in SEC filings—and the increased public awareness that goes along with them—can have real effects. Relatedly, Johnson (2020) examines whether the disclosure of one firm’s undesirable actions incentivizes its peers to avoid similar behavior. Using a U.S. government agency’s policy of disclosing violations of work-safety regulation in press releases, the study finds that firms improve compliance and experience fewer occupational injuries when they are in close geographic proximity to where the press releases were distributed to local newspapers.

In addition, the U.S. mandated the reporting of greenhouse gas (GHG) emissions for thousands of manufacturing facilities in 2010. Tomar (2021) studies this requirement, focusing on facilities for which this information was largely unavailable elsewhere. He finds that facilities reduce emissions by 7.9% following the disclosure. He also explores the mechanism for the reduction and finds evidence consistent with peer benchmarking. That is, facilities are able to assess their relative GHG performance once they can observe peer disclosures.

In 2013, the U.K. introduced a carbon reporting mandate requiring listed companies to report GHG emissions for their entire organization in annual financial reports. Jouvenot and Krueger (2020), Downar et al. (2021), and Grewal (2021) examine whether this U.K. disclosure mandate creates stakeholder pressure for firms to subsequently reduce their GHG emissions. All three studies find that the affected firms lower emissions. The estimated effect sizes differ slightly, given the different designs and control groups, but range from 10% to 18%. Exploring the mechanism, Jouvenot and Krueger (2020) pinpoint investor pressures as a potential driver of the effects, consistent with Bolton and Kacpercyk (2021). Similar to Tomar (2021), Grewal (2021) finds that emission reductions are related to peer benchmarking.

The EU has also passed several directives that mandate increased CSR disclosures. The EU Corporate Social Responsibility Directive (NFRD 2014/95/EU) requires large firms to prepare and disclose nonfinancial reports from fiscal year 2017 onward. Fiechter et al. (2020) examine the real effects around the disclosure mandate and find that firms, on average, increase their CSR activities in response to the regulation, and that they start doing so before the mandate comes into force. The effects are stronger for firms with low levels of CSR expenditures prior to the regime change. The evidence shows that a looming CSR reporting mandate could positively affect firms’ CSR and further suggests that benchmarking and peer pressure can play a role for poor CSR performers to increase their CSR activities.

Two other regulatory initiatives are the mandatory disclosure of extraction payments for oil, gas, and mining firms in the EU and in Canada. These payments represent what regulated firms pay to foreign host governments for the right to extract resources. Some argue that such payments fuel corruption in developing countries. One of the stipulated goals of the mandates is to impose transparency to curb this kind of behavior. Rauter (2020) examines the effects of the disclosure mandates and finds that disclosing firms pay higher prices for extraction rights, decrease investments, and obtain fewer extraction licenses relative to unregulated competitors. The effects are stronger for firms that face a high risk of public shaming, operate subsidiaries in corrupt host countries, or have high exposure to payments that are more vulnerable to bribery.^46^

In sum, most academic studies find that firms tend to expand and adjust CSR activities subject to disclosure requirements. One potential mechanism is benchmarking; firms want to avoid the public backlash associated with looking worse than their peers.^47^ They could also learn from their peers. However, the improvements in CSR often come at a cost (i.e., in the form of lower productivity, financial profitability, or market share). An important limitation of these studies is that their settings tend to be focused on specific disclosure items. Moreover, extant disclosure mandates were often implemented with the explicit objective of changing corporate behavior as opposed to solving information frictions in financial markets. For these reasons, it is not clear that we can generalize the results from such relatively narrow and focused settings to a broad and general CSR reporting mandate (see also Glaeser and Guay 2017).

### Effects of CSR reporting on firms’ entry and exit decisions

Based on the above evidence, it is likely that CSR reporting requirements affect firms’ cost-benefit tradeoffs not only for CSR activities and policies but also for regular operating and financing decisions. This includes decisions on whether to trade in public markets (where they typically face more extensive disclosure requirements) and whether to remain present in a specific product market. If costs rise or benefits decline following a CSR reporting mandate, firms likely adjust and could even abandon certain activities. Of course, it is unlikely that a reporting mandate alone would make firms exit core business activities, but at the margin it is conceivable that firms scale back or disinvest more peripheral operations. New firms could enter thereby changing the industry composition. Alternatively, the concentration within an industry could go up.

The firms most likely to exit (enter or expand) are those with comparatively higher (lower) costs of maintaining strong CSR performance and with high (low) reputational costs. Some of these costs may be driven by factors inherent to a firm’s business. For instance, for geological reasons, coal mines in certain areas are inherently less safe than coal mines in other areas. If it is costlier for the former mines to excel on safety and if mandated CSR metrics (partly) reveal this information, then the additional costs imposed on mine owners could lead them to reduce operations or close down after a CSR reporting mandate. We note that such real effects (e.g., making the worst polluters exit the market) are not necessarily bad and could in fact be intended by the policymakers or regulators.

The reputational costs to firms from a misalignment with stakeholders’ CSR preferences vary across firms. For instance, larger, more highly visible firms are often well-suited targets for activist campaigns and also subject to more media scrutiny subsequent to poor CSR performance, compared to smaller, lesser known firms (e.g., Watts and Zimmerman 1978). As a result, activities that are problematic or risky from a CSR perspective might shift from large to small firms. Similarly, if mandatory CSR standards apply only to SEC-registered firms, we could observe a shift of such activities from regulated to unregulated (private) firms. Consistent with this idea, Christensen et al. (2017) find that SEC-registered firms subject to mine-safety disclosure rules are more likely to shut down dangerous mine facilities than unregulated firms.

There exists evidence on market exit as a regulatory avoidance strategy in response to financial regulation (e.g., Leuz et al. 2008; Kamar et al. 2009; DeFond and Lennox 2011). Such evidence in the CSR literature is still sparse, but the concern about avoidance strategies is nevertheless relevant. For instance, Rauter (2020) shows that oil, gas, and mining firms reduce their investments in response to mandatory extraction payment disclosures, but also finds evidence of reallocation of investments from disclosing firms to firms in jurisdictions without such regulation. These shifts come at the cost of reducing drilling productivity in the host countries, suggesting that uneven disclosure regulation in an industry can distort capital allocation.

### Implications for mandatory adoption of CSR standards

Based on extant research, there are a few implications that we can derive for the real effects of a CSR reporting mandate, both at the firm level and for the economy as a whole. The implications are particularly relevant if the goal of the mandate is to drive changes in CSR and sustainability. First, assuming sufficiently specific CSR standards and proper enforcement, we expect that firms respond to a CSR reporting mandate by making real changes to their business operations, including their CSR activities and policies. The primary drivers of such real effects are probably societal or stakeholder pressures as well as peer effects and benchmarking. These forces alter firms’ cost-benefit tradeoffs for various CSR and non-CSR activities. On average, we expect firms to increase CSR activities that are covered by the reporting mandate. Moreover, we expect firms to reduce or even abandon certain business activities that are viewed as problematic (e.g., by socially responsible investors or consumers). However, we emphasize that it is extremely difficult to foresee the myriad real effects that a CSR reporting mandate could induce. Some will clearly be desired change, others will be unintended. Thus, we cannot make any statements on whether the expected changes to firms’ real activities (CSR or otherwise) are desirable from a societal perspective or even the viewpoint of investors.

Second, increasing CSR and sustainability could be desirable from a societal standpoint even when it is costly to firms such as, for instance, when these changes mitigate negative externalities. A CSR reporting mandate could make it less costly for certain stakeholders (e.g., grassroots movements, activist groups, consumer associations) to acquire and process relevant CSR information. As a result, these stakeholders would be more likely to exert pressure on corporations to address negative external effects. In response to or in anticipation of such behavior, firms could reduce their negative externalities (e.g., Amel-Zadeh and Serafeim 2018). For CSR standards to have such effects, several conditions must hold: (i) the standards need to be sufficiently specific and properly enforced (so that firms actually end up disclosing poor CSR performance or CSR omissions);^48^ (ii) poor CSR performers do not already voluntarily disclose (negative) CSR information;^49^ and (iii) to exert pressure, the relevant stakeholders must be able to understand and use the CSR information. The latter condition is not trivial. For instance, consumers are typically not experts on the environmental impact of production technologies and could have difficulties understanding the relative importance of different pollutants. If consumers do not respond in a sophisticated manner, it is hard to see how they can impose costs on firms in a way that properly reflects the relative harm of each pollutant to society, or how a CSR reporting mandate can have the desired effects on overall pollution levels, that is, can act like a Pigouvian tax.^50^

Third, one might hope that a CSR reporting mandate primarily pressures firms with poor CSR performance. However, because performance metrics are likely imperfect, even generally well-performing firms could at times report poor CSR performance, especially for metrics not fully under their control. CSR standards could therefore expose firms to additional reputational risks, in particular if stakeholders do not understand that the CSR metrics are noisy. Thus, we also expect firms with strong CSR records to make adjustments. For instance, firms with the most valuable brands have the most to lose from an exogenous CSR disaster and, hence, might react more. The counterargument is that firms for which CSR reputation risk matters the most often already are the best CSR performers and provide many CSR disclosures voluntarily. From that perspective, a mandate would affect them less.

Finally, mandatory CSR standards are unlikely to cover all firms in the U.S. economy. Financial reporting regulation is typically limited to SEC-registered firms, so most private firms are exempt. A CSR reporting mandate therefore would likely widen the gap between public and private firms, both in terms of CSR activity and CSR transparency. Indeed, one concern is that harmful CSR activities could shift from public to private firms. Similarly, public firms with high CSR risks or operating in industries with serious CSR issues could choose to go private. As a result of such reallocation, observable CSR performance by publicly traded firms could increase, yet aggregate CSR in the entire economy could improve less or even decrease. We can make a similar argument for foreign firms. If strict CSR standards unilaterally apply to U.S. firms, harmful CSR activities might shift or increase abroad, with unclear net effects on global CSR performance. In addition, compliance costs could hurt domestic industries, at least in the short run.^51^ On the flip side, well-performing foreign firms could feel attracted to a more stringent CSR regime. For instance, Boubakri et al. (2016) show that foreign firms cross-listed in the U.S. exhibit better CSR performance than their non-cross-listed peers, that the CSR performance increases at the time of the cross listing, and that the benefits are larger for firms domiciled in countries with weak institutions. These results are consistent with the notion of bonding benefits from stringent CSR standards (e.g., Lu 2021).

## Implementation issues for mandatory CSR reporting standards

In this section, we review several implementation issues that are central when establishing a CSR reporting mandate. Specifically, we discuss the process of establishing and maintaining CSR reporting standards (Section 6.1); the role of the materiality concept, including a discussion of the distinction between single and double materiality and a review of studies related to the materiality of CSR disclosures (Section 6.2); the use of boilerplate language in CSR reports (Section 6.3); and the enforcement and assurance of CSR standards and disclosures (Section 6.4).

### CSR standard-setting process

Financial reporting standards are set by the Financial Accounting Standards Board (FASB) through a process that aims to consider all relevant stakeholders’ views.^52^ To accomplish this goal, the FASB, among other things, takes requests and recommendations for new standards and holds public comment periods before issuing them. The SEC follows a similar approach to setting new (disclosure) rules. Regardless of whether mandatory CSR standards are set by a standard setter like the FASB or directly by the SEC, a similar due process will likely be applied.^53^ However, there are a few unique features of the CSR setting that, in practice, could make the CSR standard setting process different from the process for financial reporting standards.

Most importantly, the group of stakeholders that potentially use CSR disclosures is broader than for financial reporting. Of course, standard setters could limit the scope of the standards and define the target audience for CSR information more narrowly. But even when CSR standards are developed with only investors’ information needs in mind, once the information is publicly disclosed, anyone can use it. This implies that a potentially large group of stakeholders could participate in the standard setting process, and their objectives could differ substantially from those of investors. For instance, activist groups could use CSR standards to change firms’ CSR activities or policies. Investors might even share these goals, but unlike the activists, they have an economic stake in the firm and, hence, might push back on such attempts if they reduce firm value. As a result, debates over the importance and (societal) value of firms’ CSR activities will likely enter the CSR standard setting process, which is quite different from the process of setting financial reporting standards, for which the merits of the underlying transactions are typically not the focus.

To illustrate, consider the Dodd-Frank requirement that firms disclose information on the use of conflict minerals in their regulatory filings (Section 1502 of the Dodd-Frank Act). The provision for conflict mineral disclosures (CMD) was motivated by a concern that purchases of war minerals by Western companies could fuel the longstanding conflict in the Democratic Republic of Congo (DRC).^54^ Thus, the provision is a good example for an attempt to use CSR disclosures to drive change, that is, to make firms internalize negative externalities from their behavior. The SEC received more than 700 comment letters on its CMD proposal. Of these letters, 62% were from the general public and humanitarian organizations supporting the rule’s intent, and 38% were from businesses, trade and industry associations, the investment/financial community, professional audit firms, and relevant government entities.^55^ Such heavy involvement of the general public and humanitarian organizations in standard setting is uncommon for financial reporting standards or securities regulation more broadly. Moreover, it is clear from reading a subset of the comment letters that many arguments in favor of CMD are based on assessments of the severity and the costs of the conflict for people in the DRC, and not focused on the question of whether CMD are relevant to investors in their decision making.^56^ Corporate representatives and their interest groups, while generally supporting the overall objective of CMD, often point to the reporting costs they would incur as a result of the disclosure rule. Although the latter arguments are similar to those in comment letters on proposed financial accounting standards, the former arguments are not.

The CMD example illustrates the challenges of setting CSR standards. The tradeoffs are more wide-ranging and potentially more political in nature than those faced by a financial reporting standard setter. As a result, the CSR standard setting process is unlikely to center on the key economic tradeoffs with respect to providing CSR information, but instead involves value or moral judgments with respect to the underlying CSR activities. Such normative considerations are indeed appropriate if the goal is to drive change in firm behavior via CSR reporting standards. In this case, however, the CSR standard setting process requires a broader democratic legitimization, as do other regulatory interventions into firm behavior (e.g., taxes or emission limits). If the goal is more narrowly to inform investors about material CSR information, then these broader (and normative) considerations about the underlying CSR activities could result in CSR standards that are less relevant to investors’ information needs for financial decision making.

### Materiality of CSR disclosures: concepts and evidence

We begin with the definition of materiality as used in financial reporting (Section 6.2.1). We then apply it to CSR reporting and contrast it to the concept of double materiality (Section 6.2.2). We conclude with a review of the empirical CSR literature on the topic (Section 6.2.3).

#### Definition of materiality for financial reporting

Materiality is a key concept for the scope of reporting standards. The Supreme Court defines information as “material” if there is a substantial likelihood that the disclosure of the omitted fact would have been viewed by a reasonable investor as having significantly altered the “total mix” of information made available (TSC Industries, Inc. v. Northway, Inc., 426 U.S. 438 1976). Consistent with this definition, the FASB defines accounting information as material if “the magnitude of an omission or misstatement of [this] accounting information […] in the light of surrounding circumstances, makes it probable that the judgment of a reasonable person relying on the information would have been changed or influenced by the omission or misstatement” (Concepts Statement (CON) No. 2).^57^

Given this definition, a central question is who the users of the information are and for what purposes they are using the information. The FASB defines the target audience as present and potential investors and creditors who make investment, credit, and similar decisions and who have a reasonable understanding of business and economic activities (CON No. 1). In short, accounting disclosures and financial reporting target sophisticated stakeholders with a financial interest in the firm and aim to provide material, decision-relevant information to them.

#### Materiality concepts for CSR reporting: single versus double materiality

The primary issue for the application of the above financial materiality concept to CSR disclosures is that the set of relevant decision makers is broader. CSR topics are of interest to a large set of stakeholders, not just investors. From this perspective, defining and assessing materiality of CSR disclosures is more difficult, especially when the scope of the CSR standards is broad and encompasses reporting on firms’ impacts on the environment and society.

In response to this challenge, one could consider reducing the scope of the CSR standards and focus exclusively on the information needs of investors.^58^ Under such an approach (sometimes referred to as single materiality), the standards would prescribe reporting only on CSR topics that are financially material to investors. This narrow materiality concept is consistent with the goal of giving investors the information they demand or need for decision making, assuming that they care only about the financial consequences (or NPV) of firm activities. With this assumption and goal in mind, it conceptually makes sense to narrow the scope of CSR disclosures to issues that are relevant to investors’ decision making and potentially affect firms’ long-term value creation. Notably, this narrow approach excludes CSR disclosures on externalities that firms impose on society. One could make an argument that this narrow approach is essentially already prescribed by the financial materiality definition of the SEC (and the FASB). Thus, if firms are largely in compliance with the current SEC requirements, then mandated CSR standards based on the narrow approach should not produce much new information for investors. They could still provide standardization benefits or facilitate enforcement (see also Section 4.6). However, it is also possible that there exists a noncompliance issue for material CSR-related information. Moreover, there is likely to be more uncertainty about what constitutes financially material information to investors when it comes to CSR, and to be less guidance by the accounting standards or the SEC.^59^

On the opposite end of the spectrum is a materiality concept that incorporates information relevant to a wide range of stakeholders. Under this broad approach, the reporting entity considers whether and how it affects the sustainability of the systems within which it operates (e.g., the environment and society), irrespective of whether these impacts have financially material consequences for the firm.^60^ It includes reporting about externalities. The key materiality criterion is whether the CSR information is relevant to one or more stakeholders because of impacts that the firm causes, including impacts that have financially material consequences for the firm and investors (which is why this inside-out view is also called double materiality). Thus, the number of CSR reporting topics is likely large and covers a broad range of ESG issues. This materiality concept seems to be motivated by a desire to drive change through CSR reporting. The underlying idea is that broad CSR disclosures make firms internalize the (social) costs of their impacts on the environment and society and eventually lead to changes in how they operate.

Whether one chooses a narrow or a broad approach to CSR reporting depends on—among other things—normative views about the intended scope and target audience. The tradeoffs are nontrivial. As discussed in Section 5.4 and Section 6.1, a broad approach with double materiality is likely to attract external pressures from various (and potentially unforeseen) parties and also requires that standard setters apply political and moral judgments about the underlying CSR activities. For these reasons, a narrow, single materiality approach could have a certain appeal for accounting standard setters and securities regulators as it is closer to their expertise. One could also argue that a narrow approach should make it easier for reporting entities to determine what type of CSR information has to be reported and, hence, has lower compliance costs. However, even with a focus on what investors want, the boundaries of single materiality are not always clear cut. One reason is that stakeholders other than investors could care about firms’ impacts on the environment and society, and these impacts prompt them to take actions against the firm. If such (anticipated) stakeholder reactions have financial consequences for the firm, then the topic will be material to investors, and CSR disclosures that alter the financial consequences of these actions will become material to investors as well (even if the CSR issue per se seems immaterial).^61^

In addition, the assumption that investors care only about monetary returns seems unrealistic. An increasing number of investors appears to make investment decisions not only based on expected future returns, but also considers non-monetary aspects and social norms (e.g., Hong and Kostovetsky 2012). For them, the set of relevant information is much broader. For instance, an investor who disapproves of child labor wants information on a firm’s stance on this issue as well as on the use of child labor in the supply chain. Thus, maximizing shareholder welfare (rather than shareholder value; (Hart and Zingales 2017) involves information that is relevant to shareholders’ CSR preferences. Put differently, giving investors the information they want is no longer confined to financial materiality.^62^ One way to incorporate investors’ non-monetary preferences but still limit the scope of CSR reporting is to require a sufficient consensus among capital providers on the relevance of a CSR topic for it to be considered by standard setters.

On a more practical level, several additional issues arise when applying the notion of single materiality to CSR disclosures. First, CSR information is rarely expressed in monetary units, and the link between CSR activities and firm value or financial performance is, at best, tenuous (see also Section 4.1). Moreover, CSR is often long-term and intangible in nature. As a result, standard setters (ex ante) and managers (ex post) face substantial discretion in assessing the materiality of CSR topics (Matsumura et al. 2017; Amel-Zadeh and Serafeim 2018). Second, there is little history or precedent for setting materiality thresholds for CSR disclosures. New CSR reporting standards need time to evolve, and standard setters, firms, and accountants need time to learn. Yet, establishing a common materiality threshold and applying it to a broad set of firms for the very first time could substantially improve the amount and comparability of CSR disclosures relative to the status quo. Third, in financial reporting, what is material depends largely on firm-specific factors, although there clearly exist similarities in capital structures and operating activities within industries. For CSR reporting, business processes determine common CSR topics for all firms in the same industry. While some CSR topics are more generic (e.g., worker safety, labor relations), most are rather industry specific (e.g., greenhouse gas emissions for energy firms, hazardous waste for chemical firms). Thus, there is a strong industry component to CSR materiality.

Finally, financial reporting regulation often reacts to corporate scandals or financial crises, as they change what is deemed material information (Hail et al. 2018). Such changes are potentially even more pronounced for CSR reporting. CSR topics generally concern issues of broad societal interest. Societal issues can change quickly, encompass a wide variety of subjects (many of which are argued based on normative, moral, or political grounds), and sometimes are triggered by exogenous events (e.g., natural catastrophes, environmental accidents, protest movements). This fluid nature is likely more pronounced under double materiality, but could also occur under single materiality, especially if what is deemed financially material depends on stakeholder responses.

In sum, the above points suggest that the various materiality concepts for CSR disclosures pose many difficulties in practice and that the lines between them are blurred such that differences may be smaller than they appear at first.

#### Evidence on CSR materiality

Empirical studies on the determinants and economic consequences of financial materiality in general and of CSR disclosures in particular are rare (see Panel B of Table A7 in the Online Appendix).^63^ One possible explanation is that it is difficult for researchers to independently identify material information. A way to tackle the measurement problem is to consider the dollar amount of a disclosure item. In a Regulation S-K setting, Cho et al. (2012) show that firms’ decisions to provide CSR information on environmental capital expenditures (whose disclosure is required if material) are not just a function of the magnitude, as several of the reported amounts are small and arguably immaterial. Firms likely also pursue goals other than informing investors. Perhaps the disclosures are an attempt to mitigate political or regulatory pressure.^64^ However, there is also the argument that adding immaterial disclosures could increase the complexity, impose higher processing costs, and reduce the decision-usefulness of financial reports (e.g., Merton 1987; Barber et al. 2005; Dyer et al. 2017).

Dhaliwal et al. (2012) assess materiality from a user perspective, essentially arguing that what matters for users must be material. The authors focus on analysts as users and find that CSR information (proxied by the issuance of a stand-alone CSR report) is associated with lower analyst forecast errors, and that this relation is stronger in stakeholder-oriented economies and for firms in countries with more opaque financial disclosures. The results suggest that CSR materiality depends on the institutional environment. Moreover, in environments that put more weight on nonfinancial information, the materiality threshold for CSR disclosures could be lower.

Moroney and Trotman (2016) conduct an experiment to examine differences in auditors’ materiality judgment between CSR engagements and regular financial statement audits. They find that auditors apply tighter materiality thresholds for financial statements than for CSR reporting. Qualitative factors (e.g., closeness to breaching a contract or the presence of special interest groups) affect the assessment of CSR materiality. The authors argue that differences in auditor liability, lack of guidance or experience, and different justifications for CSR versus financial audit differences contribute to these findings.

Several recent studies make use of the SASB standards to examine the materiality of CSR disclosures. These studies exploit that, by design, the SASB classification distinguishes between what should be material and immaterial information to investors.^65^ For instance, Khan et al. (2016) examine the value implications of material CSR investments (according to the SASB topics) using their association with one-year-ahead stock returns. The study finds that the SASB materiality distinction captures aspects that are also reflected in stock returns. But we have to be careful when interpreting the results. They merely show that the SASB standards (or topics) roughly reflect differences between material CSR activities and irrelevant CSR activities that are already known by investors and priced by the market. The association does not imply that the information about what is material was conveyed by the SASB standards or that the materiality distinction in the standards by itself is useful to investors.^66^ In fact, the results suggest that investors were able to price firms’ CSR activities differentially even without the SASB standards being in place.^67^

Grewal et al. (2020) also examine which CSR issues are material to investors. They construct a firm-specific disclosure score of material CSR information (based on SASB topics) and find a negative association between (changes in) this score and (changes in) stock price synchronicity. One interpretation of this finding is that material CSR disclosures enable investors to incorporate firm-specific information into price. The results are stronger after the introduction of new SASB standards and for firms or shareholders for which CSR issues are more important. The authors do not find a similar relation for immaterial CSR disclosures. However, the study already finds the negative association *before* the SASB standards were issued. It is therefore not clear that the SASB materiality distinction can be (fully) credited for the results—although the analyses in changes and around the introduction of new SASB standards help mitigate these concerns.

Matsumura et al. (2017) examine climate-change risk disclosures. The SEC flagged climate risk in 2010 as potentially material under Regulation S-K, but since then has not consistently enforced its guidelines, creating ambiguity as to whether disclosure is required (see also Wasim 2019). The authors find that, in industries where information on climate risk is material according to the SASB classification, disclosing firms have significantly lower cost of capital than nondisclosing firms. In industries without a material impact of climate risk, no such gap exists. One interpretation is that the SASB materiality distinction works in identifying firms for which the disclosures are more relevant to investors. There is again the concern that the distinction captures (unobservable) firm and industry attributes that are associated with cost of capital but not controlled for by the study’s propensity score matching design.

Grewal et al. (2017) use SASB materiality to classify activist shareholder proposals as dealing with material or immaterial CSR issues. Using a difference-in-differences design, they find that proposals on both material and immaterial CSR issues are followed by respective improvements on these issues. However, the valuation implications are quite different. Tobin’s q slightly decreases in the years after immaterial CSR proposals, but it increases following material proposals.^68^ The evidence is consistent with the notion that the SASB materiality distinction matters to investors but, as the authors point out, such an interpretation hinges on the extent to which the design addresses the selection issues that arise with activist campaigns.^69^

The aforementioned studies point to the importance of distinguishing between material and immaterial information. However, they also illustrate that researchers have to be careful when they divide historically disclosed information into material versus immaterial based on standards that were only available ex post. There is the concern that the standards likely focus on topics that in the past were material. Relevant tests need to be constructed around the adoption of standards as well as with post-adoption data. The current findings leave open the important question of how well a standard setter can determine, ex ante, what CSR information will be financially material to investors in the future. Moreover, we need more research on how a broader stakeholder-oriented approach of (double) materiality affects the decision making of the various interested parties.

### Use of boilerplate language for CSR disclosures

Boilerplate disclosures in response to a CSR reporting mandate are a significant implementation concern, because firms could use them as an avoidance strategy. By boilerplate disclosures, we mean generic (mostly qualitative) disclosures that remain vague, do not provide details or metrics, and are largely uninformative (e.g., Lang and Stice-Lawrence 2015). In principle, the concern about boilerplate language arises for all qualitative disclosures (e.g., risk factor disclosures; Kravet and Muslu 2013; Campbell et al. 2014; Hope et al. 2016). In the context of CSR reporting, firms with weak incentives to report meaningful CSR information could use boilerplate language to comply with the letter but not necessarily the spirit of the standards. Consistent with this concern, textual analysis of financial reports suggests that firms often respond to new disclosure requirements by extending their boilerplate disclosures (Dyer et al. 2017). A related and more CSR-specific concern is that firms use boilerplate language for greenwashing, that is, to gloss over or detract from poor CSR performance or to provide unsubstantiated CSR claims and create more favorable impressions.

In financial reporting, boilerplate disclosures are relatively common (see, e.g., statements in SEC 1998, 2013; Higgins 2014), have been shown to exist in many countries (Lang and Stice-Lawrence 2015), and have increased over time (Dyer et al. 2017). Moreover, boilerplate disclosures likely play a role in the increasing complexity and loss of readability of corporate disclosures (e.g., Li 2008; Dyer et al. 2016, 2017).^70^ Boilerplate disclosures are also common in CSR reports (see Section 3.4).^71^ To the extent that (meaningful) mandatory CSR disclosures would be (net) costly to firms, boilerplate language is one way to mitigate these costs. Thus, a mandate is likely to increase the prevalence of boilerplate language in CSR reporting. Considering that processing information is costly and that users of financial reports often have limited attention or limited processing ability (e.g., Merton 1987; Barber et al. 2005; Barber and Odean 2008), adding boilerplate language is not innocuous, especially if it obfuscates or crowds out more relevant disclosures. With boilerplate language, CSR reports could read more like compliance documents than useful means of communication (Hoogervorst 2013).

CSR standards can limit boilerplate language by prescribing, in great detail, what information firms have to provide and how they must provide it. Much depends on the specificity of the standards. However, the more specific the standards are, the less widely applicable they are. Thus, specificity can run counter to the goal of setting standards for a broad set of firms and circumstances. In fact, given the heterogeneous nature of firms’ CSR activities, standard setters might have to build substantial discretion into the standards to make them broadly applicable (see also Section 2.4.3).

### Enforcement of CSR standards and assurance of CSR disclosures

We first outline the conditions for an effective enforcement of a CSR reporting mandate (Section 6.4.1) and then discuss the role of accounting and consulting firms as providers of CSR assurance (Section 6.4.2).

#### Effective enforcement of mandatory CSR reporting standards

A large body of academic literature suggests that enforcement is critical to the successful implementation of financial regulation and accounting standards (see Section 2.4.4). The same is likely true for CSR reporting. However, effective enforcement of CSR standards is not a given. As Peters and Romi (2013) show, even the compliance with SEC disclosure rules for environmental sanctions is low, despite the use of bright-line materiality thresholds. Of course, relative to the status quo, mandating a common set of CSR standards likely facilitates enforcement. Having specific rules helps regulators and courts to identify noncompliance.^72^

Creating an effective enforcement regime for CSR reporting poses several challenges. First, enforceable standards rely on the extent to which CSR information can be verified or audited by a third party. Given the nature of many CSR activities, verifiability of CSR disclosures is likely difficult and may differ across activities and firms. CSR metrics frequently rely on internal information, are highly subjective, and lack external reference points like price data or industry benchmarks, which would be helpful for verification.

Second, unlike financial reporting with its general ledger and double-entry bookkeeping, CSR reporting draws on many separate and ad-hoc measurement systems (O’Dwyer 2011). In some cases, CSR standards pertain to issues that go beyond the boundaries of the reporting entity (e.g., its supply chain), and the firm might not have full control over the information, which makes establishing an audit trail a challenge. Illustrating these difficulties, Kim and Davis (2016) show that out of more than 1,300 firms that are required to report, under Section 1502 of the Dodd-Frank Act, on whether their products contained “conflicted minerals,” 80% were unable to do so, and only 1% could attest with certainty that their products were conflict-free. Effective compliance essentially requires a system for CSR information similar to firms’ internal controls over financial reporting. Such a system could be costly, especially for smaller firms, as the debate on compliance with the Sarbanes-Oxley Act (SOX) shows (e.g., Coates and Srinivasan 2014; Leuz and Wysocki 2016).^73^

Third, the enforcement (or auditing) of CSR reporting requires non-accounting expertise and, in many cases, technical or scientific knowledge. Enforcement agencies that currently oversee securities regulation and financial reporting do not necessarily have these skills. Thus, extending oversight to a CSR reporting system requires additional investments in infrastructure and know-how. In light of the expertise requirements, one could consider charging different agencies with the enforcement of select disclosures (e.g., the EPA for environmental disclosures). The counterargument is that there are likely economies of scale and cost savings to firms when dealing with a single enforcement agency. Finally, regulators could consider a “comply or explain” approach for CSR standards (e.g., Ho 2017). This approach offers flexibility when the underlying activities exhibit substantial variation, as they do for CSR. Rather than forcing all firms to report in a particular way, it allows firms to deviate—for good reasons—from the CSR standards (which have more the flavor of prescribed best practices). Comply-or-explain could lower compliance costs for firms and allow them to flexibly adapt their reporting to new trends and developments. On the flip side, firms could give perfunctory explanations for noncompliance. Thus, comply-or-explain relies on well-functioning market mechanisms to penalize opportunistic noncompliance.

#### Role of accounting and consulting firms as assurance providers for CSR disclosures

In financial reporting, auditing plays a major role in assuring that firms apply and follow the accounting standards, which in turn makes financial reports more credible to investors (e.g., Beatty 1989; Blackwell et al. 1998; Willenborg 1999; Weber and Willenborg 2003; Minnis 2011). Many of the key features of CSR reporting (Section 2.3) suggest that the credibility of firms’ CSR disclosures could be low and, hence, third-party assurance would be even more important (e.g., Hasan et al. 2003; Pflugrath et al. 2011; De Meyst et al. 2018). Not surprisingly, there exists an attestation market for voluntary CSR reports, in which accountants and consultants play a role (Sìmnett et al. 2009; Casey and Grenier 2015; Michelon et al. 2019). Accounting and consulting firms differ in the way their assurance is perceived by outsiders.^74^ For instance, Pflugrath et al. (2011) conduct an experiment among financial analysts and find that the assurance value is larger for accounting firms. Michelon et al. (2019) document differences in the behavior of accountants and consultants, especially when it comes to CSR restatements. The study suggests that the behavior of accountants is shaped by their experiences in financial reporting. Moreover, Maso et al. (2020) find that the joint provision of CSR assurance and financial audits by the same audit firm improves its ability to assess going concern risk, suggesting that CSR-related information can be relevant when auditing financial reports and can improve audit quality.

The enforcement of CSR standards and the assurance of CSR reporting could be left to private parties on a voluntary basis. In this case, firms would hire their own auditors or consultants to certify their CSR disclosures. Even then, CSR reporting standards could facilitate private assurance. However, the experiences with auditing for financial reporting and with many high-profile accounting scandals suggest that voluntary private assurance is unlikely to result in effective enforcement (see also Ackers and Eccles 2015). Voluntary assurance is likely insufficient if the goal of the CSR reporting mandate is to address noncompliance with existing SEC disclosure requirements—that is, if firms withhold CSR information that is financially material to investors under the current SEC regime. We therefore believe that a combination of public and private enforcement similar to what we have for financial reporting is necessary for an effective enforcement regime (e.g., Djankov et al. 2003). Such a regime would naturally arise if mandated CSR reporting were embedded in firms’ SEC filings and came with an audit mandate.^75^

Irrespective of whether a CSR reporting mandate includes an assurance requirement or not, it likely leads to a substantial expansion of the demand for assurance services. Ioannou and Serafeim (2017) show that after the introduction of CSR reporting mandates in China, Denmark, Malaysia, and South Africa, the propensity of firms voluntarily seeking assurance of CSR reports went up. Such a shift in demand could lead to significant transition costs. In addition, a mandate could have spillover effects on the assurance market for financial and CSR reporting. Smaller, unregulated firms in particular could face difficulties and higher costs in retaining high-quality auditors or CSR consultants (see, e.g., Duguay et al. 2019 for a similar argument regarding the audit costs for private firms after SOX).

## Summary of main insights and avenues for future research

In this study, we draw on relevant academic literatures in accounting, finance, economics, and management to provide an economic analysis of a requirement for U.S. firms to report on CSR and sustainability topics. We discuss various economic consequences, including effects in capital markets, on stakeholders other than investors, and on firm behavior.

To conclude the analysis, we synthesize the main insights and briefly outline what we perceive as important questions and unresolved issues for (mandatory) CSR reporting. This summary could provide scholars with opportunities for future research. Our discussion here follows the same order as the analysis in the body of the study. That is, we begin with insights from extant academic research in accounting, finance, economics, and management that are relevant when considering mandatory CSR reporting (Section 2); review key determinants of CSR disclosure and the current state of CSR reporting (Section 3); discuss potential effects of mandatory CSR reporting standards for important stakeholders such as investors, lenders, analysts, the media, consumers, and employees, as well as for society at large (Section 4); outline likely firm responses and real effects from mandatory CSR reporting standards (Section 5); and consider important implementation issues for an effective CSR reporting mandate (Section 6).

First, to the extent that firms’ CSR disclosures provide information that is relevant to capital market participants, much of the existing literature on the effects of corporate disclosure and reporting applies. This literature suggests that more and better (CSR) information can benefit capital markets through greater liquidity, lower cost of capital, and better capital allocation. In addition, corporate disclosures can change firm behavior. Such real effects seem relevant in a CSR context, especially if the goal of mandatory CSR reporting is to influence firms’ CSR activities or to mitigate externalities of firm behavior. Real effects are more likely to follow from a reporting mandate than from voluntary disclosures. This insight implies that a CSR reporting mandate could be a tool to drive social or environmental change but could also lead to unintended consequences for firms and induce firm behavior that is undesirable to investors or society.

Prior literature shows that corporate disclosures can induce proprietary and litigation costs. Proprietary costs are one reason why firms often are reluctant to provide information voluntarily. Proprietary costs seem particularly relevant for mandatory CSR reporting, as it often involves information about firms’ business operations or production processes. This concern is especially relevant for CSR disclosures that are specific, detailed, and process-oriented, as well as for smaller firms. Litigation is one means of enforcing (disclosure) regulation and, if costly, can affect how, what, and when firms disclose. However, the relation between disclosure and litigation risk is nuanced and depends on many factors, such as the timing and content of the disclosure. We expect the relation to be equally complex for CSR reporting. From the international financial reporting literature, we can infer that the role of standards in harmonizing reporting practices is limited, particularly if managerial reporting incentives differ across firms, industries, and countries. The same should hold for CSR standards, pointing to a crucial role of the reporting infrastructure and the enforcement mechanisms in place when implementing these standards.

Although the above discussion highlights that the disclosure literature, broadly defined, has many insights to offer, one has to be careful when applying it to CSR reporting. CSR reporting is characterized by a wide-ranging, multifaceted set of topics, which are often long-term, non-monetary, and intangible in nature, as well as by a large set of users, all of which makes it quite different from financial reporting. For these reasons, it is worthwhile to revisit important empirical relations in the context of CSR reporting.

Second, our review of the CSR literature demonstrates that many of the determinants of voluntary CSR reporting are similar to those documented for financial reporting (even though there are a few specific determinants of CSR reporting practices). This result suggests that there exists substantial commonality in the economic forces that drive the two sets of disclosures. The commonality, in turn, makes it difficult for researchers to separately estimate the effects of CSR disclosures on capital markets and other outcomes. Moreover, there is substantial heterogeneity in CSR disclosures, both across and within industries. Although this evidence could be viewed as suggesting a need for harmonization and CSR standards, the observed variation likely also reflects heterogeneity in firms’ business activities, in the materiality of these activities for firms (and their investors), and in firms’ perceived costs and benefits of providing CSR information. In this sense, past reporting practices in a voluntary regime can shed light on potential compliance issues of a CSR reporting mandate. It remains an open issue to what extent CSR reporting standards can rein in the current heterogeneity in voluntary CSR disclosures and lead to harmonization effects in reporting practices within and across industries.

Third, extant literature suggests that mandatory CSR reporting has the potential to improve information to investors and other stakeholders. However, the magnitude of the resulting information effects from a CSR reporting mandate depends crucially on the extent to which firms currently withhold material CSR information. If firms largely comply with existing securities laws and already provide all material CSR-related information, then CSR standards based on financial (single) materiality should not produce much new information for investors. In this case, CSR standards could still have benefits, but they would come from standardization of reporting practices and better comparability across firms, including cost savings to firms and investors. If instead compliance with existing disclosure requirements is rather low when it comes to CSR information—as some evidence suggests—but the mandate is able to force out new and better information, then we expect capital markets to respond accordingly. In that case, CSR reporting could increase liquidity, lower the cost of capital, and improve capital allocation. However, forcing firms to provide new information likely also entails proprietary costs and heightened scrutiny by stakeholders. The former could reduce firms’ incentives to innovate with respect to CSR; the latter could have desirable and undesirable effects on firms’ behavior, which we discuss below. We lack empirical evidence on the underlying compliance question, so the magnitude of the information effects is hard to predict. Moreover, net effects of a mandate are largely an empirical matter on which we currently do not have much research.

Much of the prior evidence in the CSR literature focuses on the valuation and performance effects of CSR activities, not on CSR reporting. The key challenge therefore is to disentangle the reporting effects from the effects of the underlying CSR activities, especially when both are largely voluntary. In light of this dual selection problem, it is not surprising that studies on voluntary CSR reporting find more favorable results than studies on mandatory CSR reporting. Research on the latter is still relatively scarce and, if anything, focuses on traditional capital-market outcomes (and investors). Thus, aside from better identification that lets researchers separate the effects of CSR disclosures from CSR activities, we need more research on whether *mandated* CSR reporting mitigates information asymmetries, forces out unfavorable CSR information, generates positive spillovers, provides market-wide cost savings, or generates comparability benefits (all of which would be central to justifying a mandate). In addition, we need more research on how mandated CSR reporting affects stakeholders beyond the traditional capital-market participants because—depending on the (normative) goals of the CSR reporting mandate—it may very well be that these groups are a key, if not the primary, target audience of the CSR reporting regime. Relatedly, we also lack evidence on whether stakeholders such as consumers and employees use CSR disclosures in “traditional” outlets like stand-alone CSR reports or regulatory filings and how these potentially regulated disclosure channels compare to alternative means of communication such as websites, social media, or advertisements.

Fourth, we expect that a CSR reporting mandate induces firms to make changes to their business operations. The literature suggests that firms generally respond to mandatory CSR reporting by expanding and adjusting their CSR activities to improve CSR performance, which is typically costly to firms. Societal or stakeholder pressures as well as peer benchmarking appear to be the main explanations for these changes. Firms with a high reputation at stake and poor past CSR performance tend to respond more. If a reporting mandate results in better CSR information, we also expect managers to scale back on CSR activities that are not aligned with shareholder preferences. In this regard, it is important to note that shareholders too can have nonfinancial or CSR preferences. It is further possible that firms reduce or even disinvest business activities that are viewed negatively by stakeholders or pertain to highly sensitive CSR issues. Such real effects illustrate that mandatory CSR reporting could make it easier for influential stakeholders to exert pressure on firms to address their external effects. However, (harmful) CSR activities could also shift abroad, or to private firms if the mandate applies to SEC registrants only.

It is very difficult to predict whether the described firm responses are net positive or negative from the perspective of investors, other stakeholders, or society. Stakeholder responses to CSR information can induce firm responses (or real effects) that reduce firm value and, hence, have negative financial consequences for investors. However, CSR issues often pertain to negative externalities caused by firms’ business activities such as carbon emissions. Thus, imposing costs on firms and shareholders via a CSR reporting mandate could be intended and desirable from a societal standpoint. But it is also clear that the potential for unintended consequences from a CSR reporting mandate is large, especially if the mandate’s scope is broad (dual materiality). We need more research to better understand these tradeoffs as well as how and why firms respond to specific reporting requirements. For instance, we know little about the way firms’ real responses differ depending on their ownership, customer, and supplier structures, or about the precise causal chain from the release of CSR information to firms’ responses that result from the (anticipated) reactions of certain stakeholders to these disclosures.

Fifth, we identify several important implementation issues: the CSR standard setting process, the relevant materiality concept for CSR disclosures, the use of boilerplate language as an avoidance tool, and the enforcement or assurance of CSR standards. The process of setting CSR standards is likely shaped by societal, political, and moral debates about the underlying CSR topics themselves (rather than issues related to decision usefulness, measurement, or presentation that normally dominate the debate over financial reporting standards). Such normative considerations are indeed appropriate if the goal is to drive change in firm behavior via CSR reporting. In this case, the CSR standard setting process requires a broader democratic legitimization than what financial reporting standard setting usually has; it needs to be more akin to what we require for other major regulatory interventions into firm behavior (e.g., taxes or emission limits). However, such broader (and normative) considerations about the underlying CSR activities could result in CSR standards that are less relevant to investors’ financial decision making. How we evaluate these standards depends on whether the goal of the CSR reporting mandate is to provide material information to investors or to drive certain changes in firm behavior. At this point, we have relatively little research on CSR standard setting itself—who participates in the process, how participation differs, and which groups or arguments succeed.

Materiality of CSR information can be defined using the same principles as for traditional financial disclosures, but determining which information is material to whom is more difficult. A core problem is that the link between CSR activities and financial performance is tenuous, yet central to the definition of financial materiality. Another issue is that the relevant materiality concept depends on the goal of the CSR reporting mandate. If the goal is to “drive change” with CSR reporting, the relevant materiality concept is likely double materiality, as financial materiality almost by definition excludes reporting on firm impacts that are externalities. Focusing on financial (single) materiality makes sense if the goal is to provide relevant information to investors, but only if investors care primarily about firm value. Once investors have non-monetary preferences about CSR issues (e.g., they care about environmental impacts), even “giving investors what they want” would likely require a broader materiality concept. Thus, assessing materiality from a shareholder welfare or investor information perspective might not narrow the scope of CSR reporting by as much as one might think. Extant research on materiality of firms’ CSR disclosures often suffers from hindsight bias by identifying issues that were relevant to investors in the past. Relevant CSR issues can change quickly; hence, defining materiality in a prospective way is challenging. Moreover, showing the value relevance of CSR disclosures is not sufficient to establish materiality. Doing so requires much tighter identification. We need more research on how standard setters can ex ante determine material information to investors and other stakeholders. Relatedly, we still know little about how investors and other stakeholders specifically utilize CSR information.

Boilerplate language and greenwashing are important concerns, highlighting the difficulty of forcing firms to provide meaningful CSR information. CSR standards can play a role in reducing boilerplate language by prescribing what and how firms have to provide information, but only if the standards are specific enough (e.g., require specific metrics or numerical disclosures). However, specificity makes it more likely that the standards do not fit the circumstances of a particular firm. Setting CSR standards at the industry level can help with specificity but diminishes across industry comparability. Prior literature shows that boilerplate CSR disclosures are common, but we have little evidence on why this is the case and how it affects users’ decision making. Another interesting issue is whether firms use boilerplate language as an avoidance strategy to hide (unfavorable) CSR performance or retain proprietary information.

Finally, enforcement plays a central role if a CSR reporting mandate is to have substantive economic effects, intended or unintended. Creating an effective enforcement regime presents several challenges and requires substantial investments in infrastructure and (technical) expertise. Based on the experience with financial reporting, it makes sense to consider a combination of private assurance with public enforcement and oversight. Even absent an audit requirement for CSR information, a CSR reporting mandate likely leads to increased demand for assurance services. Central research questions in this context are whether and how enforcement improves the effectiveness of CSR standards, how the assurance of CSR reports performed by audit firms differs from the assurance provided by specialized CSR consultants, and how public and private enforcement interact with each other in the oversight of CSR reporting.

## Supplementary Information

This study is a revision and extension of an earlier, independent research report prepared for the Sustainability Accounting Standards Board (SASB) in 2018. The full report is available at https://ssrn.com/abstract=3315673. The Online Appendix accompanying the report (and referred to in this study) provides a structured overview of the CSR literature and is available at http://ssrn.com/abstract=3313793.

## Footnotes

1 Other players are the TCFD (Financial Stability Board’s Task Force on Climate-related Financial Disclosures), the CDSB (Climate Disclosure Standards Board), and the IIRC (International Integrated Reporting Council).

2 While we consider a mandate for the U.S., much of the analysis should apply to other developed market economies (although we acknowledge that countries differ in important ways, for instance, in the legal liability regimes).

3 A related term commonly used in the literature is “ESG activities.” The definitions of CSR, ESG, and sustainability are very close. See Section 2.1 for further discussion. To keep the exposition short, we often use the acronym CSR, but for us this term interchangeably refers to sustainability and ESG.

4 For instance, Liang and Renneboog (2017, p. 854) define CSR as “firm activities that improve social welfare but not necessarily at the expense of profits (or shareholder value).” A positive relation between CSR and firm value is often referred to as “doing well by doing good” (e.g., Dowell et al. 2000; Orlitzky et al. 2003; Deng et al. 2013; Flammer 2015). But others point out that the relation may run the other way, that is, firms “do good when they do well” (e.g., Hong et al. 2012; Lys et al. 2015).

5 For instance, the European Commission (2011, p. 6) defines CSR as “the responsibility of enterprises for their impacts on society,” which implies that firms “integrate social, environmental, ethical, human rights and consumer concerns into their business operations and core strategy.”

6 In case of a combined report, the term “integrated reporting” is also used (e.g., Barth et al. 2017).

7 Even with mandatory adoption of CSR standards, firms can and likely will provide voluntary CSR disclosures beyond those required under extant securities laws or the adopted CSR standards. However, the CSR reporting mandate could improve or standardize such voluntary disclosures.

8 For surveys of the CSR literature see, for instance, Renneboog et al. (2008), Kitzmueller and Shimshack (2012), Crane and Glozer (2016), Brooks and Oikonomou (2018), Grewal and Serafeim (2020), and Matos (2020).

9 An example of an exemption is the mandate for CSR investments in India, where firms that meet certain profitability and size thresholds must spend at least 2% of net income on CSR (Manchiraju and Rajgopal 2017).

10 Voluntary CSR reporting is therefore endogenous in two ways: (i) it depends on firms’ voluntary CSR activities and (ii) firms’ choices in reporting on these activities (e.g., Bouten et al. 2012).

11 There also exist situations in which disclosure can exacerbate information asymmetries, such as when only a select few sophisticated investors know how to interpret the information (e.g., Kim and Verrecchia 1994, 1997).

12 We note that better disclosure (or reporting) is hard to define and is a concept with multiple (possibly conflicting) dimensions. We use it here as a placeholder for desirable properties of corporate disclosure and reporting, in particular the usefulness of corporate information to outside investors for decision making and contracting.

13 These were two of the main arguments put forward by regulators, standard setters, and politicians for the mandatory adoption of International Financial Reporting Standards (IFRS) in many countries around the world (for overviews see, e.g., Barth 2006; Hail et al. 2010a; De George et al. 2016).

14 See Ross (1979), Grossman and Hart (1980), and Milgrom (1981).

15 Incumbent parties currently benefitting from the lack of disclosure regulation are likely to oppose such regulatory changes (Rajan and Zingales 2003).

16 In this section, we draw on a related discussion in Hail et al. (2010a) and Hail et al. (2010b) on the potential adoption of IFRS in the U.S.

17 The reason why reporting standards allow for discretion is to let managers convey their private information about firm performance to corporate outsiders (e.g., Watts and Zimmerman 1986). At the same time, managers could use discretion to obfuscate economic performance and achieve personal goals.

18 For instance, EC Regulation No. 1606/2002, which introduced mandatory IFRS reporting for most firms traded on regulated markets in the EU in 2005, required each EU member state to take appropriate measures to ensure compliance. However, because the timing and degree of enforcement differed substantially by EU country, the ensuing capital-market outcomes differed as well (Christensen et al. 2013).

19 We draw these insights on CSR reporting practices of U.S. firms from a SASB (2016) report on the state of CSR disclosures in SEC filings as well as our own (independent) analysis of a proprietary dataset that the SASB gathered from regulatory filings and provided to us. See Christensen et al. (2018), Section 3.2.

20 See Kitzmueller and Shimshack (2012) for an extensive discussion on why this perspective is too narrow. One could think of Friedman’s position as a starting point, like the Modigliani and Miller irrelevance theorem, that only holds under certain conditions (such as perfect competition), which likely are violated in practice.

21 CSR could also reduce agency costs by better matching certain principals and agents or play a role in providing intrinsic incentives to management and employees (Besley and Ghatak 2005). See also Section 4.5.3.

22 One way of mitigating the concern about unobserved growth opportunities is to study performance effects around growth shocks that are unanticipated by managers. For instance, Lins et al. (2017) find that firms engaging in CSR fared better through the (arguably unanticipated) financial crisis. But this study does not disentangle CSR activities and reporting about them.

23 See also Barth et al. (2017), who study—among other things—how firm value is associated with the quality of firms’ integrated reporting after the mandate was introduced in South Africa.

24 Other return-based tests of CSR suggest: (i) the performance of CSR investments is generally negative or zero (e.g., Galema et al. 2008; Renneboog et al. 2008; Di Giuli and Kostovetsky 2014; Larcker and Watts 2020) unless CSR activities are considered material to investors (Khan et al. 2016); (ii) a firm’s inclusion in a CSR index is accompanied by no or slightly positive excess returns, while the removal from the index coincides with substantial negative returns, pointing to the certification role of the index provider (e.g., Doh et al. 2010; Becchetti et al. 2012; Ramchander et al. 2012); and (iii) acquiring firms with a strong CSR focus realize higher merger announcement returns (Deng et al. 2013).

25 Cornell (2021) reviews evidence that certain CSR characteristics or ESG exposures are priced in returns like an insurance premium, offering lower returns because they provide a hedge, for instance, against climate risk.

26 For firms that wait with the adoption of CSR standards until the mandate, by revealed preference, the cost-benefit tradeoff is likely negative. See Daske et al. (2013) or Christensen et al. (2015) for similar arguments regarding voluntary IFRS adoption. Such net costs can be the result of high implementation efforts, high operating costs, or low perceived benefits.

27 Integrated reporting requires firm disclosures on intellectual, human, social, and natural capital—all topics that can broadly be described as CSR.

28 CSR information is not necessarily beneficial in terms of information asymmetry and, hence, liquidity, and could lead to more disagreement among investors. For instance, Christensen et al. (2021) find that ESG disclosures generally exacerbate rather than resolve ESG rating disagreement and that this disagreement is associated with higher price volatility and a lower likelihood of external financing.

29 Becchetti et al. (2015) find a positive relation between CSR and idiosyncratic volatility. Mishra and Modi (2016) find a negative relation of CSR effort with idiosyncratic risk, but only in the presence of marketing capability, that is, the ability to convert marketing effort into future sales. Humphrey et al. (2012) find no such relation in their sample.

30 For instance, Hart and Zingales (2017) show that (prosocial) shareholder preferences affect optimal production decisions when externalities of these decisions are not perfectly separable. They drive a wedge between maximizing firm value and shareholder welfare (the latter of which also reflects shareholder preferences). In Pástor et al. (2020), investors’ CSR preferences can induce firms to engage in CSR activities through the valuation and cost of capital effects that come with these preferences.

31 Similar arguments can be made for the matching of managers and shareholders (see Besley and Ghatak 2005).

32 In contrast, Stellner et al. (2015) do not find beneficial effects of superior CSR performance using credit default swaps.

33 Competitors are another recipient group of CSR reporting. A concern here is that standardized CSR reporting could require firms to disclose proprietary information. The issue of proprietary costs could be more pronounced for CSR disclosures because these disclosures go beyond aggregate financial measures, are often directly related to a firm’s core operations, and might be very targeted and (industry) specific (see Section 2.4.1). In the extreme, a CSR disclosure mandate could lead a firm to alter its behavior (see Section 5).

34 A related phenomenon in financial accounting was the “label adoption” of IFRS, in which firms (voluntarily or mandatorily) adopted IFRS without materially changing their underlying reporting practices. See Daske et al. (2013).

35 For instance, Siano et al. (2017) examine the 2015 Volkswagen emissions scandal (“Dieselgate”) from such a perspective and argue that, in the extreme, external greenwashing can induce internal changes in the organization that are diametrically opposed to the publicly promoted CSR ideals.

36 For instance, Habel et al. (2016) show that how customers perceive firms’ markups regarding the (additional) costs of CSR activities affects their overall view of firms’ CSR engagement and, hence, subsequent outcomes like customer loyalty. CSR standards could shape customer perceptions by making such markups more transparent.

37 For instance, Darendeli et al. (2021) exploit the expansion of CSR ratings coverage from the Russell 1000 index to the Russel 2000 firms and find that newly covered supplier firms with low CSR ratings experience reductions in corporate customers and supply-chain contracts.

38 Conversely, an increase in the reliability of CSR reports could strengthen the insurance-like benefits from CSR, lead to a reduction in third-party litigation, and ease the resolution of such proceedings (e.g., Godfrey et al. 2009).

39 The cost of gathering and processing information without CSR standards could make investors with CSR preferences avoid certain industries altogether, rather than pick the best-CSR firms within the industry. This effect could make it harder for investors to diversify their holdings and reduce risk sharing in the economy.

40 Not all stakeholders are interested in overall CSR performance; some focus on certain aspects or specific CSR dimensions. For instance, survey evidence by Amel-Zadeh and Serafeim (2018) suggests that professional investors are mainly interested in CSR information that is financially material to investment performance.

41 See Hombach and Sellhorn (2019) for a similar analysis of the real effects of targeted transparency regulation.

42 There is related evidence in economics on the role of quality disclosures (see survey by Dranove and Jin 2010). Prominent examples are Jin and Leslie (2003) studying the mandatory disclosure of restaurant hygiene scorecards and Dranove et al. (2003) analyzing health care report cards.

43 According to Van der Lugt et al. (2020), there were approximately 350 different mandatory reporting requirements related to ESG issues in place across the world in 2020. For a list and description of these regulatory initiatives see https://www.carrotsandsticks.net/.

44 Mandatory disclosure settings alleviate certain selection issues because the decision to disclose is not made by the individual firm (see Section 4.2.1). However, regulation is rarely enacted in a vacuum. Mandatory CSR reporting as well as engagement in CSR activities are, in part, a result of societal pressure. Thus, it is difficult to disentangle the effects of mandatory reporting from changes in the underlying CSR activities that would have occurred absent regulation. See Leuz and Wysocki (2016) for further discussion of this issue.

45 In a similar vein, Barth et al. (2017) find positive relations between CSR disclosure quality and future realized operating cash flows and investment efficiency for firms that are subject to an integrated reporting mandate on the Johannesburg Stock Exchange in South Africa. They argue that potential real effects (and not capital-market effects like improved liquidity) are the main drivers of the observed relations. However, because the analysis is limited to post-adoption data, the authors cannot attribute the results to the mandate, but rather show variation in *voluntary* CSR reporting after integrated reporting has become a requirement.

46 Hombach and Sellhorn (2018) find negative stock price reactions upon the announcement of similar regulation in the U.S, consistent with mandatory extraction payment disclosures being perceived as costly to firms.

47 For instance, exploiting a regression discontinuity design, Cao et al. (2019) find that the passage of close-call shareholder-sponsored CSR proposals is followed by similar CSR proposals and investments by peer firms.

48 Empirically, Boiral (2013) shows for a small set of energy and mining firms that 90% of significant negative events (e.g., major spills or conflicts with local residents) were not properly discussed in these firms’ CSR reports.

49 One could argue that the unraveling result of Ross (1979), Grossman and Hart (1980), and Milgrom (1981) contradicts this condition. However, the unraveling mechanism might not apply to voluntary CSR disclosures because (i) several of the recipients are arguably less sophisticated than investors (e.g., consumers, politicians), (ii) CSR disclosures contain a substantial proprietary component (e.g., Verrecchia 1983), (iii) it is not clear to information users that managers actually have the relevant CSR information (e.g., Dye 1985), or (iv) the relevant information may not always be verifiable.

50 Consumers’ inability or high costs of understanding complex issues is an argument for implementing so-called “command and control” regulation. For instance, the U.S. Federal Aviation Administration has wide-ranging powers to regulate civil airline safety. An alternative solution would have been to require airlines to disclose safety information and leave the ultimate decision of airline safety to consumers. Yet, in this highly complex area, disclosure regulation is unlikely to be a cost-effective substitute for “command and control” regulation.

51 The pressure of moving less costly but also less efficient technologies abroad could be short-lived and eventually benefit domestic industries if they get a head start in the development and marketing of innovative technologies of the future (e.g., development of the autonomous car, applications of artificial intelligence).

52 The FASB defines stakeholders in a broad way as “those who prepare and use financial reports.” See: https://www.accountingfoundation.org/jsp/Foundation/Page/FAFSectionPage&cid=1351027541293.

53 For instance, the SASB follows a similar due process to the FASB and SEC, including having consultation and comment periods before issuing sustainability reporting standards.

54 By requiring CMD, Congress intended to “bring greater public awareness of the source of issuers’ conflict minerals and promote the exercise of due diligence on conflict mineral supply chains … [thereby] inhibit[ing] the ability of armed groups … to fund their activities by exploiting the trade in conflict minerals … [and] put pressure on such groups to end the conflict” (SEC Release No. 34-67716; File No. S7-40-10).

55 We collected these data from ELM Consulting Group International LLC (2011), retrieved on January 31, 2018.

56 Comment letters were also submitted by investors and organizations representing investors that identify as socially responsible. They often argue that the CMD will help them assess firms’ social responsibility efforts.

57 The FASB definition of materiality recently was in flux, but after several proposed amendments, it reverted to the language used in Concept Statement No. 2. The FASB definition is very similar to and consistent with the materiality notion used by the SEC, the Public Company Accounting Oversight Board (PCAOB), and the American Institute of Certified Public Accountants (AICPA).

58 For instance, the SASB is a standard setter that follows such a narrow, investor-focused approach (SASB 2017b).

59 Another factor is that, in practice, material misstatements (e.g., outright lies) are probably taken more seriously than material omissions, and typically the lack of “material” CSR disclosure would fall under the latter category. See, for instance, Wasim (2019) for evidence suggesting lack of disclosure of material information on climate change risks.

60 The Non-Financial Reporting Directive (NFRD 2014/95/EU) adopted by the EU as well as the GRI standards are examples of disclosure regimes in which the impact of companies’ activities on society or the environment can establish materiality (i.e., they follow a double materiality concept).

61 A good example for such (anticipated) stakeholder action is the environmental impact of plastic packaging or containers (like drinking straws). Plastic packaging generally makes up a small portion of product costs, but increasingly has become a concern to consumers and environmental groups, leading them to take action (e.g., boycott firms). These stakeholder actions could in turn be financially material to firms.

62 Interestingly, at a roundtable held at Harvard Law School on June 19, 2017, most legal scholars present expressed their belief that information speaking to such non-monetary investor objectives already falls under the current materiality definition by the Supreme Court (SASB 2017a).

63 We note that in this section (and the literature in general), CSR materiality refers to a single materiality approach, as extant studies examine standard capital market outcomes to assess the financial materiality of CSR disclosures.

64 Along those lines, Marquis and Qian (2014) find, for a sample of Chinese firms, that in provinces with more developed government institutions and monitoring mechanisms, firms’ CSR disclosures become less symbolic and more substantive.

65 The SASB identifies *material* CSR topics by industry through a predefined process that includes empirical evidence from data analysis, feedback from industry working groups, a public comment period, and the final review and approval by an independent Standards Council (see Khan et al. 2016). Through this process, the SASB has identified 26 CSR issues of varying materiality across 77 industries (SASB 2021a, 2021b).

66 See Holthausen and Watts (2001) for a similar criticism of the use of value relevance research to justify the design of financial reporting standards.

67 Khan et al. (2016) find that portfolios of high-CSR-materiality firms outperform low-CSR-materiality firms. The same relation does not hold for immaterial CSR information. Importantly, the SASB materiality distinction was not available to investors for large parts of the sample period, since the SASB released its provisional standards in 2012 while the tests in Khan et al. (2016) are based on stock returns from 1992 to 2013.

68 In a related study, Schopohl (2017) also examines the determinants and consequences of material and immaterial shareholder CSR proposals. The author shows that certain investor groups are better than others at seeking out financially material CSR issues, and firms with material CSR issues and concerns in the past are more likely to be targeted by activist investors.

69 Broadly consistent with the archival evidence based on the SASB definition of materiality, Guiral et al. (2020) conduct an experiment and find that investors who explicitly consider CSR performance are able to price material CSR disclosures. Mispricing only occurs for immaterial CSR disclosures when subjects (i.e., MBA students taking the role of investors) are not prompted to explicitly assess CSR issues.

70 Guay et al. (2016) suggest that firms might use voluntary disclosures to mitigate the effects of increased financial statement complexity.

71 For instance, a report by the Business and Human Rights Resource Centre (2017) examines disclosures related to the U.K. Modern Slavery Act of 2015, designed to combat modern slavery and human trafficking. It finds widespread use of boilerplate language under this regulation to formally comply with the law.

72 Shleifer (2005) makes this point convincingly for the enforcement of disclosure regulation by courts. However, enforcement cannot address discretion that is deliberately built into the standards to give firms flexibility (Leuz 2007). For this reason, reporting incentives matter a great deal. See also Section 2.4.3.

73 Poor internal controls can also give rise to costs in the form of misreporting and associated consequences in capital markets (e.g., Ge et al. 2017).

74 Firm characteristics like the composition of the board or whether the firm employs a Chief Sustainability Officer can also affect the choice between accounting firms or consulting firms (e.g., Peters and Romi 2015), as do country-specific factors like whether firms are domiciled in stakeholder-oriented economies (e.g., Sìmnett et al. 2009).

75 In such a regime, CSR assurance services could also be subject to public audit oversight. See Gipper et al. (2020) for evidence on the effects of public audit oversight for financial reporting.
